# In Silico Evaluation of the Thr58-Associated Conserved Water with KRAS Switch-II Pocket Binders

**DOI:** 10.1021/acs.jcim.2c01479

**Published:** 2023-02-28

**Authors:** Renne Leini, Tatu Pantsar

**Affiliations:** School of Pharmacy, Faculty of Health Sciences, University of Eastern Finland, Yliopistonranta 1C, 70210 Kuopio, Finland

## Abstract

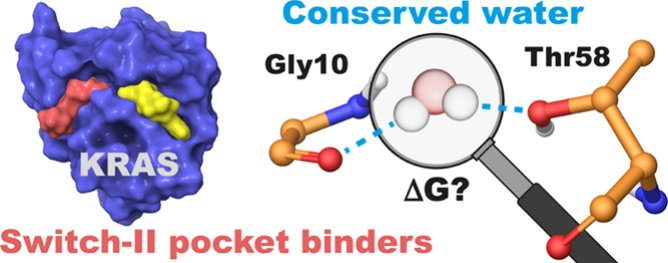

The KRAS switch-II pocket (SII-P) has proven to be one of the most successful tools for targeting KRAS with small molecules to date. This has been demonstrated with several KRAS(G12C)-targeting covalent inhibitors, already resulting in two FDA-approved drugs. Several earlier-stage compounds have also been reported to engage KRAS SII-P with other position 12 mutants, including G12D, G12S, and G12R. A highly conserved water molecule exists in the KRAS SII-P, linking Thr58 of switch-II and Gly10 of β1 sheet. This conserved water is also present in the cocrystal structures of most of the disclosed small-molecule inhibitors but is only displaced by a handful of SII-P binders. Here, we evaluated the conserved water molecule energetics by the WaterMap for the SII-P binders with publicly disclosed structures and studied the water behavior in the presence of selected inhibitors by microsecond timescale molecular dynamics (MD) simulations using two water models (total simulation time of 120 μs). Our data revealed the high-energy nature of this hydration site when coexisting with an SII-P binder and that there is a preference for a single isolated hydration site in this location within the most advanced compounds. Furthermore, water displacement was only achieved with a few disclosed compounds and was suboptimal, as for instance a cyanomethyl group as a water displacer appears to introduce repulsion with the native conformation of Thr58. These results suggested that this conserved water should be considered more central when designing new inhibitors, especially in the design of noncovalent inhibitors targeting the SII-P.

## Introduction

Mutated *KRAS* is considered one of the key targets in cancer drug discovery.^1^ The success of covalent inhibition of KRAS(G12C) to its switch-II pocket (SII-P) (reviewed in^2,3^) is exemplified by the first clinically approved KRAS-targeting drugs **sotorasib** and **adagrasib**.^4,5^ This success has cultivated the currently ongoing efforts to target the SII-P of other KRAS mutants beyond G12C, including G12D,^6^ G12S,^7^ and G12R.^8^ However, a number of other approaches toward direct KRAS targeting with small molecules, such as targeting SI/SII (switch-I/switch-II) site, have mainly resulted in lower-affinity compounds.^9−14^ Therefore, SII-P appears to offer the most promising approach to tackle this oncoprotein with small-molecule inhibitors that have been reported to date.

**Figure 1:**
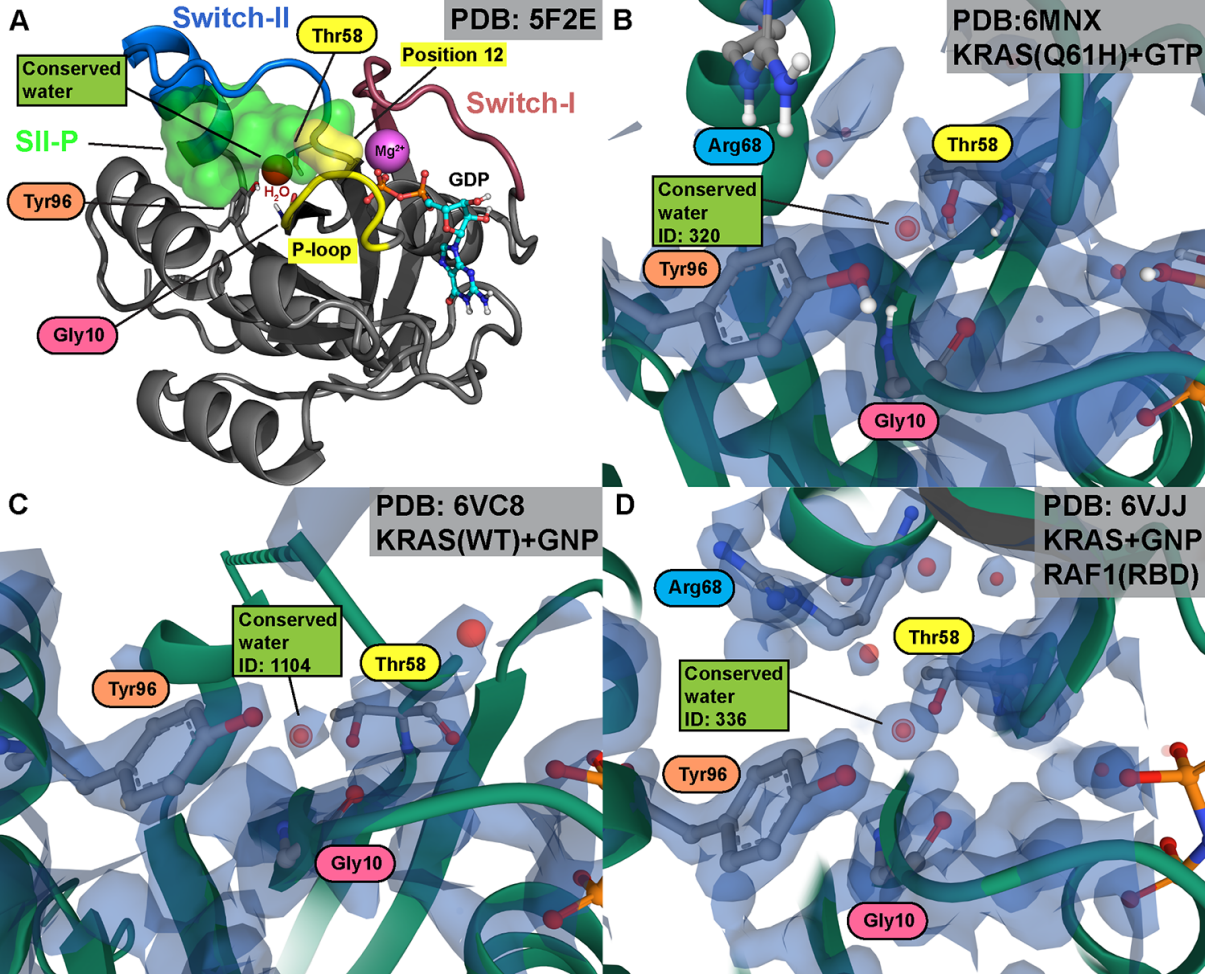
Structure of the KRAS G-domain and location of the Thr58-associated conserved water. (A) KRAS structure with selected highlighted areas (PDB ID: 5F2E).^16^ SII-P (green transparent surface) is enclosed by switch-II (blue cartoon) and reachable from position 12 (yellow transparent surface) located in the P-loop (yellow cartoon). The conserved water (red sphere) resides in SII-P next to Thr58, Gly10, and Tyr96 (shown in sticks). This water appears in numerous KRAS crystal structures, as highlighted here with examples of electron density maps of (B) KRAS Q61H bound to GTP (PDB ID: 6MNX),^17^ (C) KRAS WT bound to GNP (PDB ID: 6VC8),^18^ and (D) KRAS bound to GNP in complex with effector protein RAF1 (RBD) (PDB ID: 6VJJ).^19^ Electron density (blue transparent surface) displayed at 2Fo – Fc σ = 1.5.

The SII-P is very enigmatic in nature, as it is enclosed by the highly dynamic switch-II region (Figure 1A).^15^ Interestingly, a conserved water molecule, located between the β1 sheet and switch-II in SII-P, is commonly found in KRAS structures (Figure 1A; Table S1). In this location, the conserved water is usually connected with hydrogen bonds (H-bonds) to Gly10 of β1 sheet and to the side chain hydroxyl of Thr58 of switch-II. Occasionally, a H-bond from the water to Tyr96 hydroxyl is also observed. This highly conserved water is found in this location regardless of KRAS configuration, including wild-type KRAS (Figure 1B), different oncogenic mutants (Figure 1C), and KRAS–effector protein complexes (Figure 1D). The water in this location, when there is no SII-P binder present, is associated with high energy (Figure S1). The conserved water is also present with the majority of SII-P binding KRAS inhibitors, with only a few exceptions.

Targeting water offers opportunities to boost ligand binding affinity, and it has proven instrumental in several known cases.^20−27^ In the current drug discovery climate, targeting water is gaining increased traction as water energies can be predicted by computational approaches.^28,29^ One such method is WaterMap,^30,31^ which is based on inhomogeneous solvation theory.^32^ WaterMap evaluates the hydration site energies (relative to the bulk water) based on a short molecular dynamics (MD) simulation where nonsolvent heavy atoms are restrained. Here, we set out to analyze the nature of this Thr58-associated conserved water in more detail with the published SII-P binders. We applied the WaterMap methodology and microsecond timescale molecular dynamics (MD) simulations with two different water models (TIP3P and TIP4P)^33^ to enlighten the potential role of this water in KRAS-targeted drug discovery. Our results reveal the high-energy nature of this conserved water in the presence of SII-P binders and its suboptimal replacement with the few compounds that displace this water to date. These findings offer important insights into the conserved water and will aid the ongoing and future ligand/drug design efforts toward the most promising druggable KRAS pocket to date.

## Results

First, we present the results of the structural and WaterMap analyses of the conserved water with all of the SII-P binders with publicly available structures, where the individual KRAS mutants targeting SII-P binders (G12C, G12D, G12S, and G12R) are collected in separated sections. In addition, the KRAS(G12C) SII-P binders are divided into two sections, based on their conserved water-displacing ability. Finally, we show the results of the microsecond timescale simulations of the selected six SII-P binders (G12C, G12D, and G12S). The 2D structures of the SII-P binders can be found in Table S2.

### Covalent KRAS(G12C) Binders with Conserved Water

**Figure 2:**
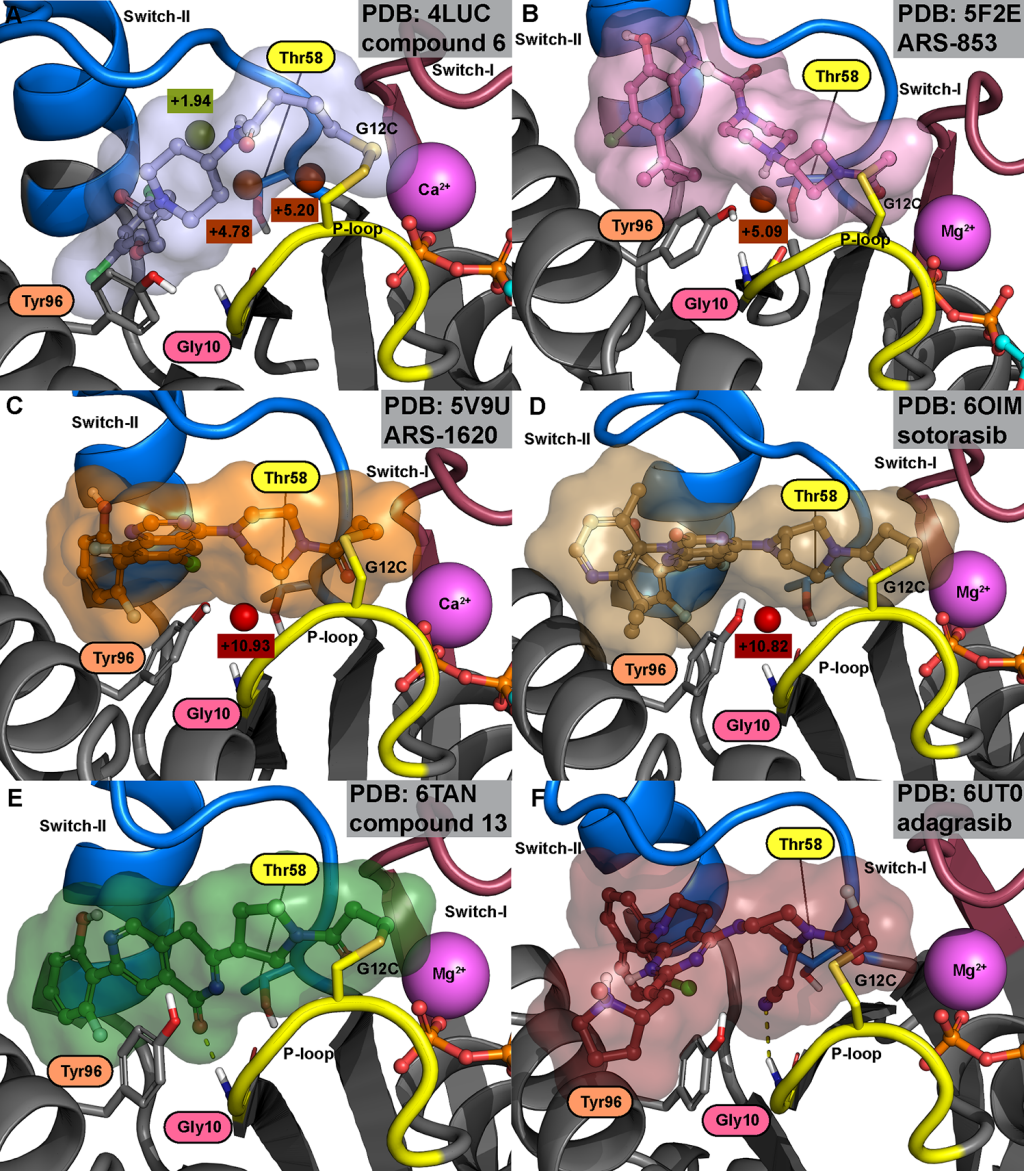
KRAS(G12C) inhibitors with and without the Thr58-associated conserved water. (A) Compound **6** of the first described series of G12C inhibitors by Ostrem et al. has adjacent hydration sites (Δ*G* = +1.93 and +5.20 kcal/mol) next to the conserved water (Δ*G* = +4.78 kcal/mol). (B) **ARS-853**, the first cell active G12C inhibitor, appears with an isolated Thr58-associated hydration site (Δ*G* = +5.09 kcal/mol). (C) **ARS-1620**, the first in vivo active G12C inhibitor, displays a high-energy conserved hydration site (Δ*G* = +10.93 kcal/mol). (D) **Sotorasib**, the first FDA-approved G12C inhibitor, exists with high-energy conserved water (Δ*G* = +10.82 kcal/mol). (E) Bayer compound **13** displaces the conserved water and forms a H-bond with Gly10. However, it is suboptimal to the native conformation of Thr58 (occupancy of 0.5 in the shown configuration; see also Figure S5). (F) **Adagrasib**, the second FDA-approved G12C inhibitor, displaces the conserved water by a cyanomethyl group, simultaneously promoting a non-native flipped configuration of Thr58. The conserved water in (A)–(D) is also found in the crystal structures (see Table 1).

The SII-P was discovered by Ostrem et al.^34^ The team reported the first set of covalent KRAS G12C binders that were occupying this pocket. In total, 12 KRAS(G12C) inhibitor cocrystal structures were released, including disulfide-, vinyl sulfonamide-, and acrylamide-based covalent inhibitors (Table 1). Most of these early inhibitors (8 of 12) appear in a distinct switch-II configuration, where switch-II resides in a more open conformation, and the conserved water site is distorted (Table 1, Figure S2). Notably, in five of these crystal structures, the ligand is found to occupy the pocket only in one of the monomers (while three KRAS proteins are forming the asymmetric unit) (Table 1). In addition, in one of these structures, the switch-II is disordered (PDB ID: 4M21)^34^ (Figure S2). Therefore, due to their distinct configuration (Figure S3), we excluded these eight structures from our WaterMap analysis. The other four structures all display the Thr58-associated conserved water in their structures (Table 1). WaterMap simulations suggest that two of the structures (PDB IDs: 4LUC; 4LV6)^34^ appear with neighboring adjacent hydration site(s) (Figures 2A and S4), while the other two (PDB IDs: 4LYH; 4M22)^34^ exhibit isolated conserved water (Figure S4). The estimated energies of the conserved water are higher with the structures of isolated hydration sites (WaterMap Δ*G*: +9.68 and +11.37 kcal/mol for isolated hydration sites; +4.78 and +7.17 kcal/mol for the conserved hydration site with adjacent hydration sites). Generally, the conserved hydration site energy with SII-P binders is higher than that observed for structures without an SII-P binder (Figure S1).

**Table 1:** Thr58-Associated Conserved Water Energies among KRAS(G12C) SII-P Binders

PDB ID	reference	ligand (name in the original publication)	resolution (Å)	conserved water in the crystal structure yes/no/yes^c^	WaterMap Δ*G* (kcal/mol) [+adjacent hydration sites if available]
4LUC	Ostrem et al.^34^	compound **6**	1.29	yes	+4.78 [+1.94; +5.20]
4LV6	Ostrem et al.^34^	compound **4**	1.50	yes	+7.17 [+1.70]
4LYF	Ostrem et al.^34^	compound **8**	1.57	no	N/A^d^
4M21	Ostrem et al.^34^	compound **11**^b^	1.94	no	N/A^d^
4LYJ	Ostrem et al.^34^	compound **9**	1.93	no	N/A^d^
4LYH	Ostrem et al.^34^	compound **9**	1.37	yes	+9.68
4M1O	Ostrem et al.^34^	compound **7**^b^	1.57	no	N/A^d^
4M1S	Ostrem et al.^34^	compound **13**^b^	1.55	no	N/A^d^
4M1Y	Ostrem et al.^34^	compound **15**^b^	1.49	no	N/A^d^
4M1W	Ostrem et al.^a^	Name unavailable	1.58	no	N/A^d^
4M1T	Ostrem et al.^34^	compound **14**^b^	1.70	no	N/A^d^
4M22	Ostrem et al.^34^	compound **16**	2.09	yes	+11.37
5F2E	Patricelli et al.^16^	**ARS-853**	1.40	yes	+5.09
5V6S	McGregor et al.^35^	compound **1**	1.70	yes	+10.37
5V6V	McGregor et al.^35^	compound **3**	1.72	yes	+9.38
5V71	Lu et al.^36^	compound **1**	2.23	yes	+10.95
5V9L	Zeng et al.^37^	**1_AM**	1.98	yes	+10.06
5V9O	Zeng et al.^37^	**3_AM**	1.56	yes	+9.18
5V9U	Janes et al.^38^	**ARS-1620**	1.38	yes	+10.93
5YXZ	Swaminathan et al.^a^	**JBI484**	1.70	yes	+10.88
5YY1	Swaminathan et al.^a^	**JBI739**	1.69	yes	+10.87
6B0V	Hansen et al.^39^	**ARS-107**	1.29	yes	+8.50
6B0Y	Hansen et al.^39^	**ARS-917**	1.43	yes	+11.24
6N2J	Fell et al.^40^	compound **4**	1.80	yes	+9.27
6N2K	Fell et al.^40^	compound **12**	1.72	yes	+6.86
6OIM	Canon et al.^41^	**sotorasib** (AMG 510)	1.65	yes	+10.82
6P8W	Shin et al.^42^	compound **2**	2.10	yes	+9.26
6P8X	Shin et al.^42^	compound **5**	2.11	yes	+8.73
6P8Y	Shin et al.^42^	compound **6**	2.31	yes	+8.57
6P8Z	Shin et al.^42^	compound **1**	1.65	yes	+10.92
6PGO	Lanman et al.^43^	compound **2**	1.60	yes	+10.66
6PGP	Lanman et al.^43^	compound **9**	1.50	yes	+10.97
6T5B	Kettle et al.^44^	compound **25**	1.37	yes^c^	displaced (methyl)
6T5U	Kettle et al.^44^	compound **29**	1.72	yes	+9.81
6T5V	Kettle et al.^44^	compound **26**	1.31	yes	+11.17
6TAM	Mortier et al.^45^	compound **3**	1.64	no	displaced (carbonyl)
6TAN	Mortier et al.^45^	compound **13**	1.16	no	displaced (carbonyl)
6USX	Fell et al.^46^	compound **7**	2.27	yes	+9.97
6UT0	Fell et al.^46^	**adagrasib** (MRTX849)	1.94	no	displaced (cyanomethyl)
6USZ	Fell et al.^46^	compound **12a**	2.03	no	displaced (cyanomethyl)
7MDP	Davies et al.^47^	**GNE-2897** (noncovalent; +antibody)	1.96	yes^c^	+6.24 [+3.92; +0.30]
7RP3	Davies et al.^47^	**GNE-1952** (+antibody)	2.00	yes	dewetted region^e^
7RP4	Davies et al.^47^	**GNE-1952**	2.15	yes	+10.73
7R0M	Weiss et al.^48^	**JDQ443**	1.61	yes	dewetted region^e^
7R0N	Weiss et al.^48^	compound **2**	1.20	no	displaced (phenyl)
7R0Q	Weiss et al.^48^	compound **3**	1.95	yes	+10.13
7OO7	Kettle et al.^49^	compound **28**	1.48	yes	+9.20
7O70	Kettle et al.^49^	**AZD4625**	1.18	yes	+12.04
7O83	Kettle et al.^49^	compound **23**	2.38	no	displaced (distal methyl in a heterocyclic ring system)
7YCC	Imaizumi et al.^50^	compound **5c**	1.79	yes	+9.32
7YCE	Imaizumi et al.^50^	compound **7b**	1.80	yes	+9.26
8DNI	Zhu et al.^51^	WO2020/028706A1 “compound **I-1**”	1.50	yes	+8.53
8DNJ	Zhu et al.^51^	WO2020/178282A1 “compound **76**”	1.81	no	displaced (distal methyl in a heterocyclic ring system)
8DNK	Zhu et al.^51^	WO2020/085493A1 “compound **6**”	2.23	yes	+10.40
8AFC	Bröker et al.^52^	compound **12**^b^	2.41	yes	+6.48 [+2.28; +4.07; −0.28]
8AFB	Bröker et al.^52^	**BI-0474**	1.12	yes	+5.36 [+5.38; +5.17; +4.63; +2.56] (alternative Lys16 conformation: +6.68 [+4.59; +3.61; +7.05])

a Unpublished.

b Ligand is cocrystallized in the SII-P only in one monomer of the asymmetric unit.

c Unassigned electronic density at the water location.

d N/A = not available due to different switch-II configurations.

e Dewetted region = WaterMap predicted the highly unfavorable region for water (Δ*H* > 8kcal/mol) that remained unoccupied by any atoms during the simulation.^31^

The first-reported selective KRAS(G12C) inhibitor with notable cellular efficacy was **ARS-853**.^16^ Interestingly, WaterMap predicts the lowest energy (Δ*G*: +5.09 kcal/mol) for this hydration site from all of the isolated hydration sites with all of the publicly available KRAS SII-P cocrystallized complexes (Figure 2B). This is due to the piperazine of **ARS-853** being optimally placed to offer the protonated nitrogen toward the water, resulting in low enthalpy of the conserved hydration site (Δ*H*: −0.18 kcal/mol). Nevertheless, the entropy of the hydration site is still estimated as high, resulting overall in a high energy hydration site (Δ*G*: +5.09 kcal/mol).

Indeed, if the Thr58-associated conserved water is present in the structure, the more recent KRAS(G12C) SII-P binders reported after **ARS-853** tend to contain only isolated conserved high-energy water (a total of 32 published cocrystal structures). The only exceptions are where the conserved water appears with adjacent hydration sites, such as the lower-affinity fragment **GNE-2897** reported by Genentech (PDB ID: 7MDP),^47^ cocrystallized together with an antibody, and Boehringer–Ingelheim’s fragment compound **12** and **BI-0474** (PDB IDs: 8AFC, 8AFB)^52^ (Table 1; Figure S6). In addition to this, the **BI-0474** structure (PDB ID: 8AFB)^52^ also displays two conformations of Lys16 (normal and shifted), with a probability of 0.5 for both, which in turn has an impact on the WaterMap-predicted hydration site pattern (Table 1; Figure S6). WaterMap suggests that all of these several distinct compounds with the isolated hydration site exhibit truly a high-energy hydration site in this location (Δ*G*: >8.50 kcal/mol). Notably, these include the first in vivo active **ARS-1620** (Δ*G*: +10.93 kcal/mol) (PDB ID: 5V9U)^38^ (Figure 2C) and optimized compounds, including Novartis’s **JDQ443** (high-energy dewetted region) (PDB ID: 7R0M)^48^ (Figure S6; Table 1), AstraZeneca’s **AZD4625** (Δ*G*: +12.09 kcal/mol) (PDB ID: 7O70)^49^ (Figure S6), and the clinically approved **sotorasib** (Δ*G*: +10.82 kcal/mol) (PDB ID: 6OIM)^41^ (Figure 2D).

### Covalent KRAS(G12C) Binders Displacing the Conserved Water

**Figure 3:**
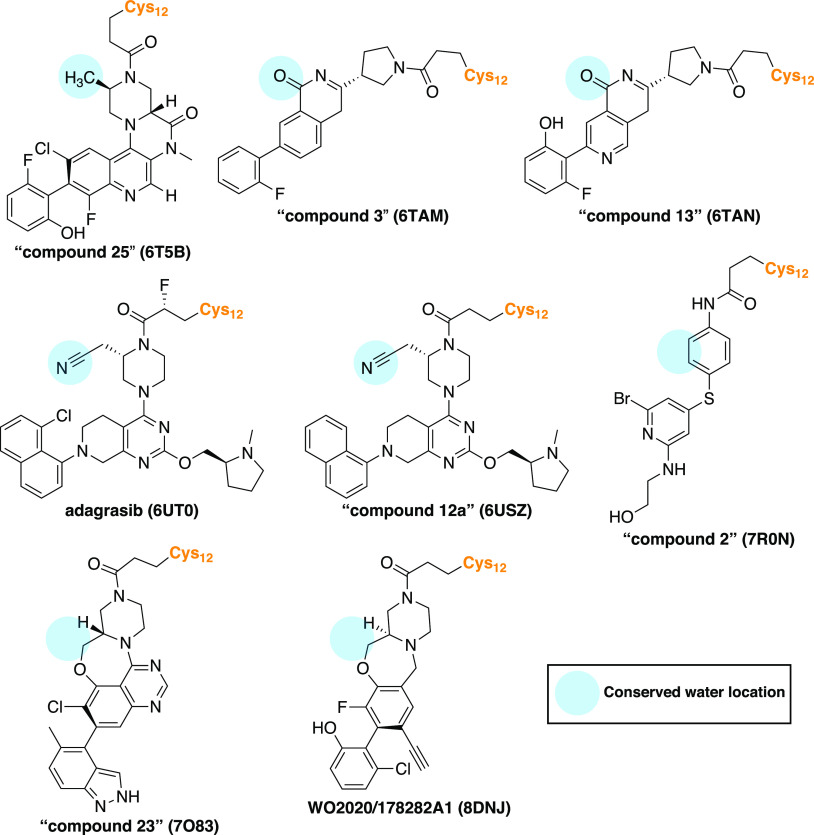
KRAS(G12C) inhibitors that have displaced the Thr58-associated conserved water. The conserved water location is highlighted with a blue circle. PDB IDs for the cocrystal structures are given in parentheses. Three-dimensional (3D) visualizations of their WaterMap results are provided in Figures 2E–F and S7.

A total of eight structures have been published where the Thr58-associated conserved water is displaced (Figure 3, Table 1). Kettle et al. were the first to publicly disclose a cocrystal structure of an SII-P binder, compound **25**, displacing the conserved water (PDB ID: 6T5B)^44^ (Figures 3 and S7). The introduction of a chiral (*R*-)methyl group to displace the conserved water, in turn, promoted the compound’s bioactive conformation and significantly boosted the potency. Interestingly, in the 6T5B structure, a positive (electron) density in the location of the conserved water is observed based on the Fo – Fc difference map (Figure S8), leaving a possibility for water existence in this location. Still, WaterMap does not predict a hydration site in this location (Figure S7). Bayer scientists introduced compounds with a carbonyl group of isoquinolinone, replacing the water and forming a H-bond with Gly10 (PDB IDs: 6TAM; 6TAN)^45^ (Figures 3, 2E, and S7). Mirati scientists applied a cyanoethyl strategy in their series to displace the conserved water and to interact with Gly10 (PDB IDs: 6UT0; 6USZ)^46^ (Figures 3, 2F, and S7). Introducing the cyanoethyl boosted the potency by 300-fold, and this cyanoethyl is present in **adagrasib**, which was approved by FDA in December 2022. Novartis compound **2** (PDB ID: 7R0N)^48^ displaces the conserved water by introducing its phenyl ring on the site (Figures 3 and S7). Finally, a distal methyl in a heterocyclic ring system occupies the conserved water site with AstraZeneca’s compound **23** (PDB ID: 7O83)^49^ and Amgen’s WO2020/178282A1 (PDB ID: 8DNJ)^51^ (Figure 3, Figure S7). However, a similar scaffold in this location is not a guarantee for the conserved water displacement (PDB IDs: 7OO7; 7O70)^49^ (Table 1, Figure S6). The native conformation of Thr58 is altered with all of the four compounds that are reaching deeper into the pocket and engaging Gly10 with an electronegative atom (fully shifted in 6UT0, 6USZ;^46^ ambiguous in 6TAM, 6TAN)^45^ (Figure S5). Analogous conformation distortion of Thr58 is not observed with the nonpolar displacers that are not reaching as deep into the pocket (6T5B;^44^7R0N;^48^7O83;^49^8DNJ^51^) (Figures 3 and S7).

### KRAS(G12D) Binders with the Conserved Water

**Figure 4:**
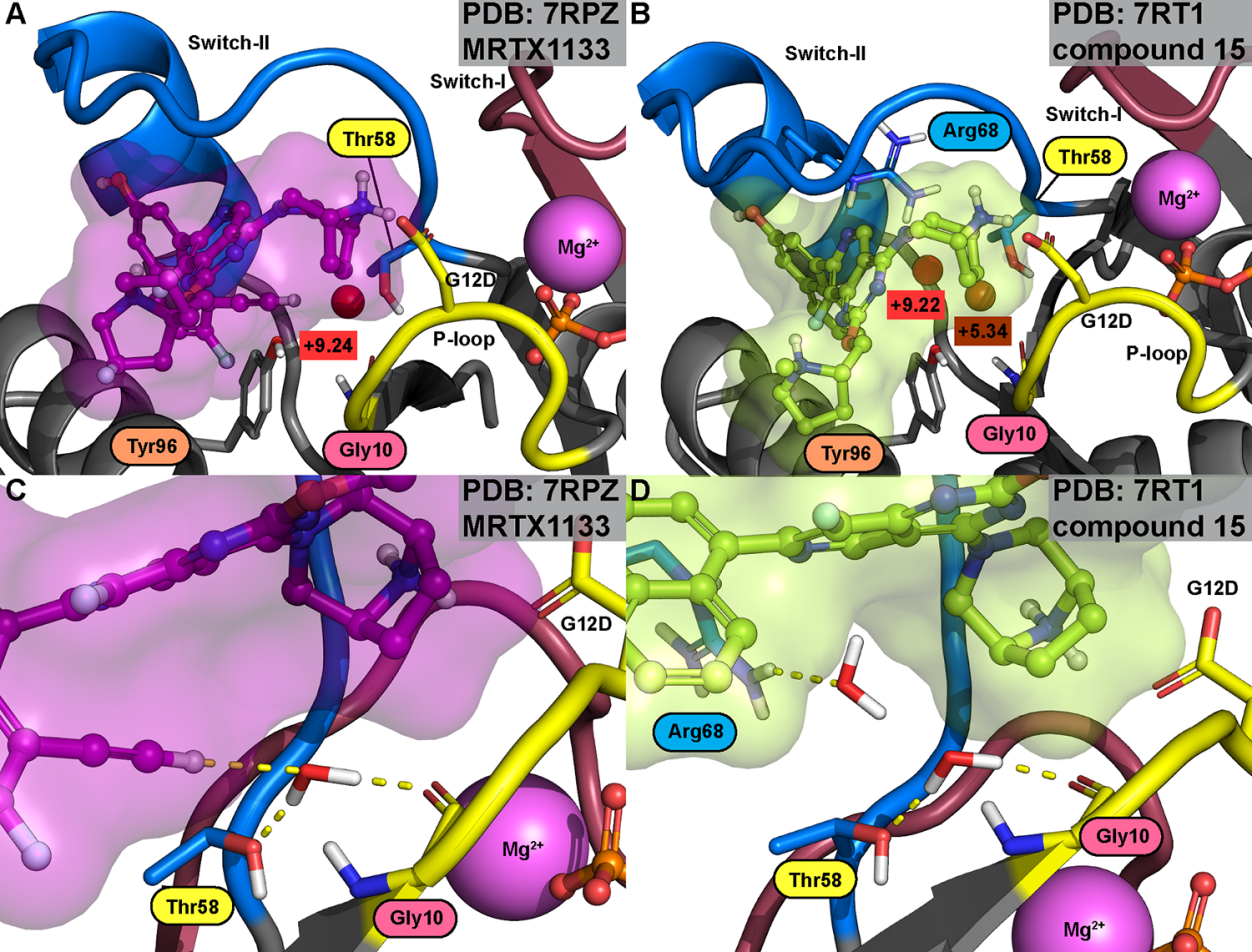
KRAS(G12D) inhibitors exist with Thr58-associated conserved water. (A) **MRTX1133** displays an isolated high-energy conserved hydration site (Δ*G* = +9.24 kcal/mol). (B) Compound **15**, which exhibits lower affinity compared to **MRTX1133**, displays an additional adjacent high-energy hydration site (Δ*G* = +9.22 kcal/mol) next to the Thr58-associated conserved water (Δ*G* = +5.34 kcal/mol). (C) Based on the crystal structure, 8-ethynyl of **MRTX1133** interacts with the conserved water (PDB ID: 7RPZ).^6^**(D)** Adjacent water is also found in the cocrystal structure of compound **15** (PDB ID: 7RT1).^6^

Mirati scientists were the first to publicly disclose SII-P-binding KRAS(G12D) inhibitors.^6^ These compounds were partially based on their successful G12C targeting **adagrasib** scaffold. Conversely, with the G12C targeting compounds, the reported potent G12D binders lack the conserved water-displacing cyanomethyl group, which was observed not to be beneficial for these noncovalent binders. Initially, Wang et al. published six structures (PDB IDs: 7RPZ; 7RT1; 7RT2; 7RT3; 7RT4; 7RT5),^6^ which all display the Thr58-associated conserved water (Table 2; Figures 4 and S9). Moreover, Wang et al. reported that the 8-ethynyl of **MRTX1133** is actively participating in the conserved water interaction network (Figure 4). Here, however, our WaterMap analysis failed to demonstrate this as a beneficial effect, as the lower energy of the conserved water was not associated with **MRTX1133**. Compared to the other inhibitors of the Mirati G12D-series, we observed that the flagship compound **MRTX1133** and compound **36**, which both contain 8-ethynyl, employ this moiety to displace the adjacent hydration sites next to the conserved water that are present with the other inhibitors of the series (Figures 4 and S9). These adjacent waters are also present in the crystal structures (Figure S10). This kind of adjacent hydration site is also observed, for instance, with low-affinity (*K*_D_ > 1500 μM) fragment **GNE-2897**, which is cocrystallized in the presence of the switch-II-stabilizing antibody (PDB ID: 7MDP),^47^ as well as with some of the first-generation G12C inhibitors with lower activity (PDB IDs: 4LV6; 4LUC)^34^ (Table 1, Figures 2, S4, and S6).

**Table 2:** Thr58-Associated Conserved Water Energies among KRAS(G12D) SII-P Binders

PDB ID	reference	ligand	resolution	reported *K*_D_ (nM)	conserved water in the crystal structure yes/no/yes^c^	WaterMap Δ*G* (kcal/mol) [+adjacent]
7RPZ	Wang et al.^6^	**MRTX1133** (GDP)	1.30	∼0.0002^a^	yes	+9.24
7RT1	Wang et al.^6^	compound **15**	1.27	0.8^a^	yes	+5.34 [+9.22]
7RT2	Wang et al.^6^	compound **25**	1.59	n/a	yes	+7.54 [+6.96; +3.25]
7RT3	Wang et al.^6^	compound **24**	1.56	n/a	yes	+6.86 [+7.01; +3.16]
7RT4	Wang et al.^6^	compound **5B**	2.10	3500^a^	yes	+4.62 [+5.71]
7RT5	Wang et al.^6^	compound **36**	1.29	n/a	yes	+9.02
7T47	Hallin et al.^53^	**MRTX1133** (GCP)	1.27	n/a	yes	+9.54
7EW9	Mao et al.^54^	**TH-Z816**	2.13	2580^b^	yes	+9.71
7EWA	Mao et al.^54^	**TH-Z827**	2.25	6520^b^	yes^c^	+8.73
7EWB	Mao et al.^54^	**TH-Z835**	1.99	670^b^	yes	+8.29

a Surface plasmon resonance assay.

b Isothermal titration calorimetry assay; n/a, data not available.

c Unassigned electronic density at the water location.

In contrast to the covalent inhibitors binding exclusively to inactive GDP-bound KRAS(G12C), **MRTX1133** is also capable of binding active GTP-bound KRAS(G12D) (PDB ID: 7T47).^53^ The Thr58-associated conserved water appears in the **MRTX1133** structures regardless of the bound nucleotide (PDB IDs: 7RPZ (GDP); 7T47 (GTP-analogue GCP)). WaterMap predicts a comparable energy value for the conserved water of these structures (+9.24 and +9.54 kcal/mol) (Figure S9), suggesting a negligible effect of the γ-phosphate (of GTP) on this hydration site.

All three G12D SII-P binder structures reported by Mao et al. exist with an isolated conserved high-energy hydration site (Table 2).^54^ Here, the ligand with the highest activity displays energy (slightly) lower than or similar to (+8.29 kcal/mol) the less active compounds of the series (+8.73 and +9.71 kcal/mol) (Table 2 and Figure S9). Interestingly, the cocrystal structure containing **TH-Z827** has no water assigned in this site in the crystal structure (PDB ID: 7EWA),^54^ but the Fo–Fc difference map displays positive density at this location (Figure S11), potentially indicating the presence of water in the site, which is also supported by WaterMap (Table 2, Figure S9).

### Covalent KRAS(G12S) Binders with the Conserved Water

**Figure 5:**
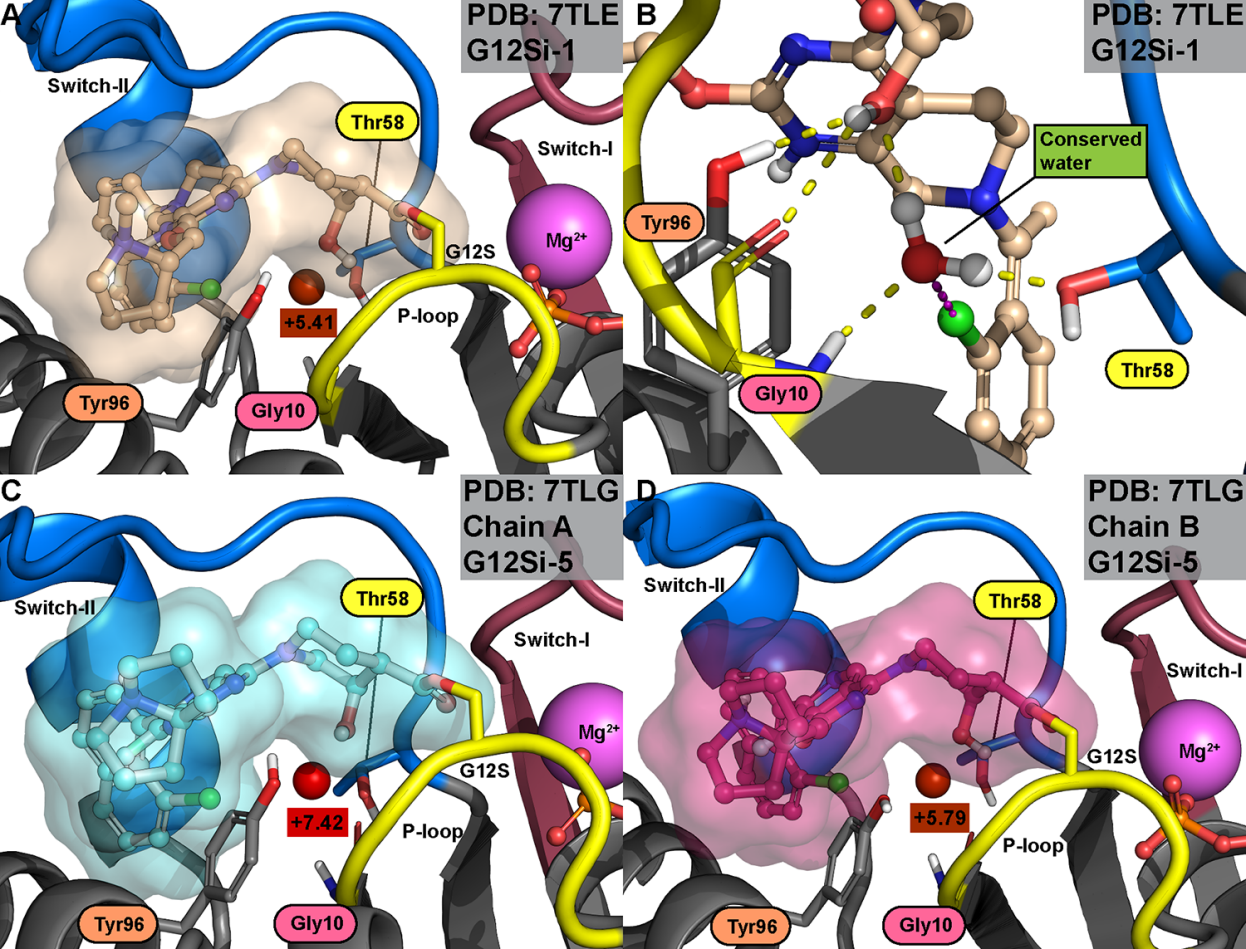
KRAS(G12S) inhibitors interact with the Thr58-associated conserved water in their covalently bound state. (A) **G12Si-1** displays a single isolated conserved water (Δ*G* = +5.41 kcal/mol). (B) Interactions of the conserved water when in complex with **G12Si-1** in the energy-minimized crystal structure. H-bonds are displayed with yellow dashed lines, and halogen bonds are displayed with magenta dashed lines. Also, the other H-bonds of the hydroxyl group of **G12Si-1** (to Gly10 and Tyr96) are shown. (C) With chain A of **G12Si-5**, a higher energy (Δ*G* = +7.42 kcal/mol) is estimated for the conserved hydration site. (D) With chain B of **G12Si-5**, comparable energy to **G12Si-1** for the conserved water is observed (Δ*G* = +5.79 kcal/mol). (See Figure S12 for the conformation differences of Lys16 in C and D.) The conserved water is also found in the crystal structures (see Table 3).

Recently, Shokat’s group reported the first covalent inhibitors of KRAS(G12S) with a β-lactone warhead reacting to the mutant serine residue (PDB IDs: 7TLE; 7TLG).^7^ Interestingly, these inhibitors present a hydroxyl group toward the conserved water after the covalent reaction has taken place (Figure 5). Among all SII-P binders with an isolated hydration site, WaterMap predicts the lowest energy for these G12S inhibitors (only surmounted by **ARS-853** (Table 1)) (Table 3). The lower energy of this water is explained by the low enthalpy due to the H-bonding interaction with the ligand, where the hydroxyl acts as an acceptor (**G12Si-1**: Δ*H* = +0.36, −*T*Δ*S* = +5.05 kcal/mol; **G12Si-5** chain B: Δ*H* = +1.26, −*T*Δ*S* = +4.53 kcal/mol; **G12Si-5** chain A: Δ*H* = +4.24, −*T*Δ*S* = +3.18 kcal/mol). Here, we noted a discrepancy in WaterMap-predicted energies on the conserved water site between chains A and B of **G12Si-5** (PDB ID: 7TLG) (Figure 5C,D). This discrepancy is explained by the slightly shifted orientation of the Lys16 toward the conserved water, which alters the environment (Figure S12).

**Table 3:** Thr58-Associated Conserved Water Energies among KRAS(G12S) SII-P Binders

PDB ID	reference	ligand	resolution	reported *K*_i_/*k*_inact_	conserved water in the crystal structure yes/no/yes^a^	WaterMap Δ*G* (kcal/mol)
7TLE	Zhang et al.^7^	**G12Si-1**	1.99	97 μM/0.41 min^–1^	yes	+5.41
7TLG	Zhang et al.^7^	**G12Si-5**	1.80	26 μM/6.4 min^–1^	yes	+7.42 (chain A)
						+5.79 (chain B)

a Unassigned electronic density at the water location.

### Covalent KRAS(G12R) Binders with Displaced Water

**Figure 6:**
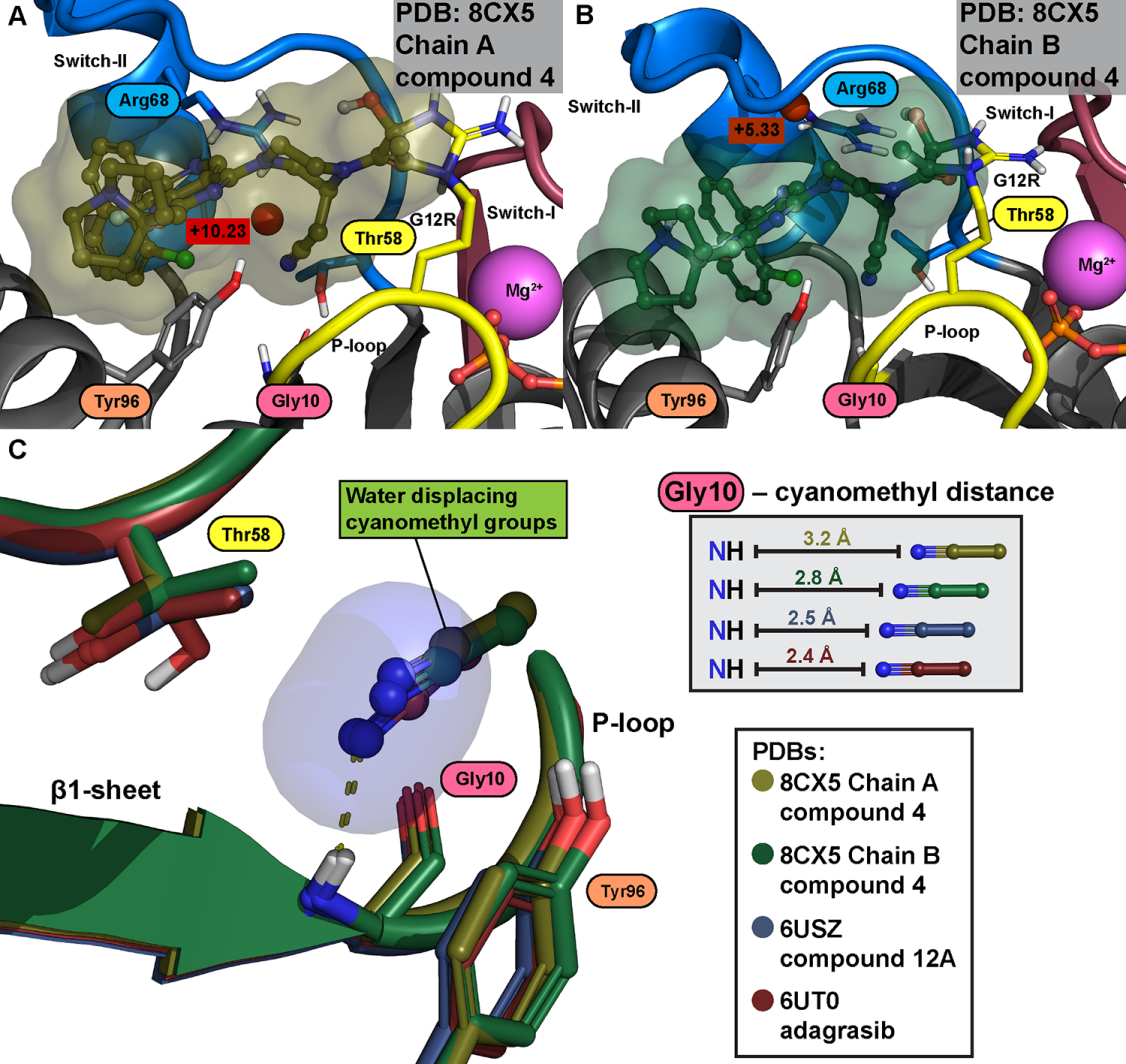
KRAS(G12R) inhibitor compound 4 displaces the Thr58-associated conserved water. (A) In chain A, an adjacent high-energy hydration (Δ*G* = +10.23 kcal/mol) next to the displaced conserved site is observed. (B) In chain B, a flipped Thr58 configuration is observed (probability of 0.71) and no adjacent hydration sites to the displaced conserved water are predicted by WaterMap. However, between the switch-II loop and Arg68 side chain, a unique high-energy hydration site is observed (Δ*G* = +5.33 kcal/mol). (C) Superimposition of all published cyanomethyl displacers reveals that G12C inhibitors **adagrasib** (red structure) and compound **12a** (gray) are better-positioned and capable of introducing H-bonding interaction with Gly10 (yellow dashed lines), while cyanomethyl of G12R inhibitor compound **4** resides further away (gold and green). With chain A of 8CX5, which displays the longest cyanomethyl distance, Thr58 is not flipped.

Shokat’s group also reported the first covalent inhibitors of KRAS(G12R) with α,β-diketoamides as reacting warheads toward the mutated arginine residue.^8^ The one publicly available cocrystal structure reveals that the conserved water is displaced by the cyanomethyl group of compound **4** (Figure 6A,B; Table 4). Interestingly, WaterMap suggests that a high-energy hydration site appears adjacent to this site with chain A of 8CX5 on the switch-II side (+10.23 kcal/mol). Notably, this is not observed with the G12C inhibitors with cyanomethyl (PDB IDs: 6USZ; 6UT0)^46^ (Figures 2F and S7). This is likely due to different conformations of Arg68 and Thr58, which in a different configuration with G12R inhibitor compound **4** vacates a suitable space for a water molecule in that location with chain A (Figure 6C). However, with chain B, no water molecules are observed in the conserved site or adjacent to it. Interestingly, a similar altered conformation of Thr58 (where the hydroxyl is flipped away from the ligand and Gly10), which was observed with cyanomethyl containing G12C binders **adagrasib** and compound **12a** (6USZ; 6UT0) (Figure S13, Figure S5), is preferred only in chain B of G12R inhibitor compound **4** (probability of 0.71) (Figure 6B,C). Here, WaterMap also predicts a high-energy hydration site (Δ*G* = +5.33 kcal/mol) next to Arg68, between its side chain and backbones of Qln61 and Glu63, which is not observed with the other inhibitors due to different Arg68 configurations (Figure 6B).

**Table 4:** Thr58-Associated Conserved Water Energies among KRAS(G12R) SII-P Binders

PDB ID	REF	ligand	resolution	conserved water in the crystal structure yes/no/perhaps^a^	WaterMap Δ*G* (kcal/mol) [+adjacent]
8CX5	Zhang et al.^8^	compound **4**	1.72	no	displaced (cyanomethyl) [+10.23] (chain A)
					displaced (cyanomethyl) (chain B)

a Unassigned electronic density at the water location.

### Molecular Dynamics Simulations Reveal the Conserved Water Behavior in Longer Timescales with Selected SII-P Binders

**Figure 7:**
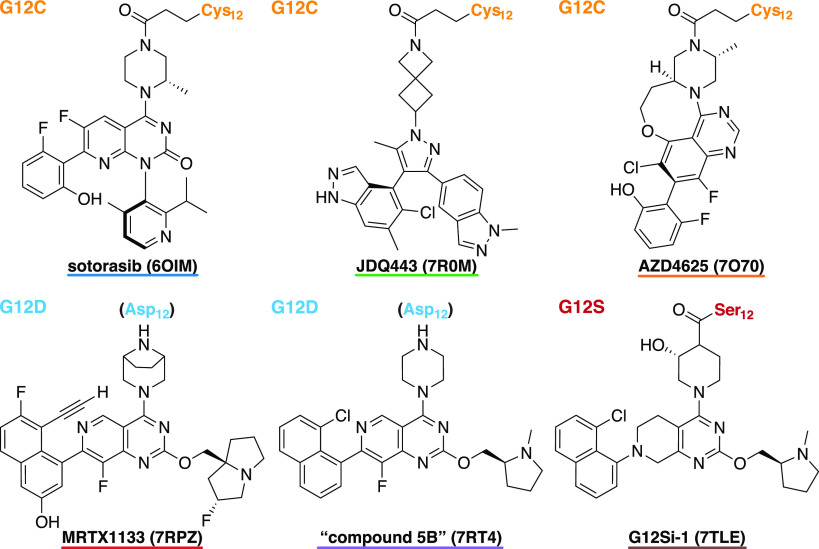
Selected KRAS SII-P binders for the microsecond timescale molecular dynamics simulations. KRAS(G12C) targeting inhibitors: **sotorasib**, **JDQ443** and **AZD4625**; KRAS(G12D) targeting inhibitors: **MRTX1133** and compound **5B**; KRAS(G12S) targeting inhibitor: **G12Si-1**. PDB IDs for the cocrystal structures are given in parentheses.

The WaterMap predictions are highly dependent on the input coordinates of the protein, thereby the method is unable to consider potential conformational changes of the closely residing residues. Therefore, we applied microsecond timescale molecular dynamics (MD) simulations^55^ to investigate the water behavior on the Thr58-associated conserved water site in the presence of selected SII-P binding inhibitors in more detail. Here, we focused our analysis on six systems: three G12C, two G12D, and one G12S system (Figure 7). We selected from the KRAS(G12C) SII-P binders the clinically approved **sotorasib** (PDB ID: 6OIM),^41^ Novartis compound **JDQ443** (PDB ID: 7R0M),^48^ and AstraZeneca compound **AZD4625** (PDB ID: 7O70).^49^ All of these KRAS(G12C) inhibitor complexes contain the conserved water in their crystal structures, while WaterMap simulations predicted high energy or even dewetted cavity (for **JDQ443**) on this hydration site (Table 1, Figures 2 and S6). We chose **MRTX1133** (PDB ID: 7RPZ)^6^ and another lower-affinity compound of the Mirati series compound **5B** (PDB ID: 7RT4),^6^ which contained adjacent waters in the crystal structure and in the WaterMap hydration site analysis, from the G12D structures (Table 2, Figures 2 and S9). Finally, we selected the covalent inhibitor **G12Si-1** (PDB ID: 7TLE)^7^ designed by Shokat et al. from the G12S structures; for this structure, WaterMap suggested clearly lower (but still high) energy for the water compared to other systems (Table 3, Figure 5).

First, we paid our attention to the protein behavior and ligand stability in the simulations. The highest fluctuations of switch-II residues of KRAS are observed with **sotorasib** and **AZD4625**, whereas **MRTX1133** and **JDQ443** show the most stable protein in this region (Figure S14). The systems with compound **5B** and **G12Si-1** fall in between the other in their switch-II behavior (Figure S14). All ligands are relatively stable during the simulations based on ligand root-mean-squared deviation (RMSD) values (Figure S15). Generally, higher flexibility is perceived with **G12Si-1** compared to other ligands. However, in one of the replicas of compound **5B**, linked to its individual higher RMSD values, we observed a partial dissociation of the compound near the conserved water site (Figure S16). There is generally no difference between the used (TIP3P and TIP4P) water models on the ligand and protein stability.

**Figure 8:**
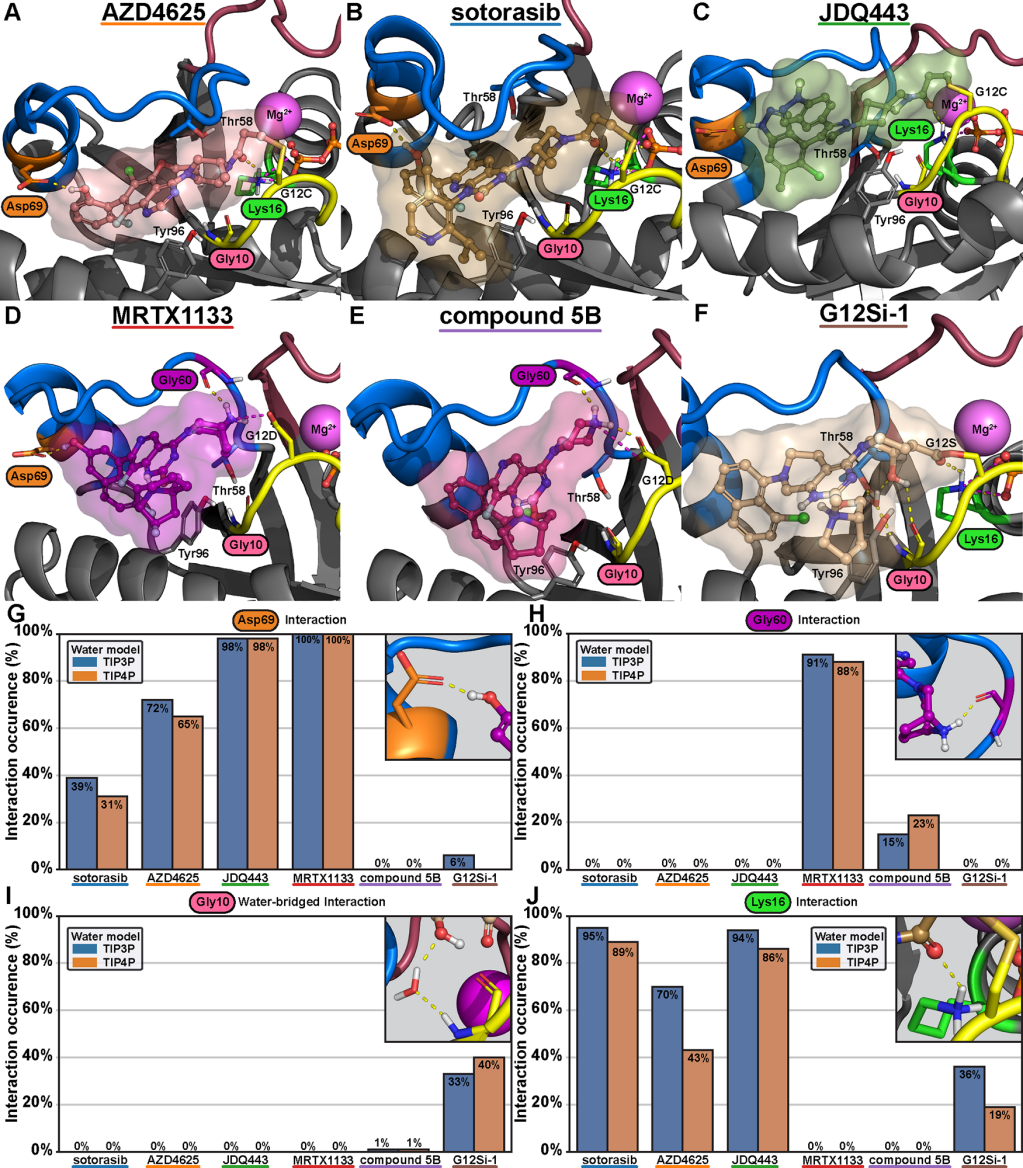
Selected protein–ligand interactions of the KRAS SII-P binders in the microsecond timescale molecular dynamics simulations. Representative snapshots of the MD simulations (A–F) highlighting the binding modes of the compounds and the locations of the discussed interactions. Frequencies of the selected interactions: (G) Asp69, (H) Gly60, (I) Gly10, and (J) Lys16. Data in (G)–(J) consist of 10 μs for each system, which was analyzed every nanosecond (=10,000 data points for each system). The more complete ligand-specific interaction frequencies for individual ligands are provided in Figures S17 and S18.

We moved on to investigate the observed protein–ligand interactions in the simulations. The most stable switch-II-associated compounds, **MRTX1133** and **JDQ443**, exhibit extremely stable (98–100%) interactions with Asp69, which is located on the opposite site of the pocket to position 12 (Figures 8, S17, and S18). **Sotorasib** and **AZD4625** also interact with Asp69, but this is partially water-mediated, reflecting the elevated switch-II flexibility associated with these compounds. When compound **5B** is compared to **MRTX1133**, it lacks the Asp69 interactions and displays clearly diminished direct interaction with Gly60 of switch-II (88–91 and 15–23%, respectively) (Figures 8, S17, and S18). These missing interactions offer a putative explanation of compound **5B** instability and experimentally observed lower binding affinity compared to **MRTX1133** (Table 2).^6^ The only compound engaging actively in interactions with the conserved water is **G12Si-1**. The compound maintains the water-bridged interaction with Gly10 from its hydroxyl group with 33–40% frequency in the simulations (Figures 8, S17, and S18). **G12Si-1** interactions to Lys16, which is in the proximity of the conserved water site, are clearly lower (19–36%) compared to what is observed for other covalent inhibitors (43–95%) (Figures 8, S17, and S18).

**Figure 9:**
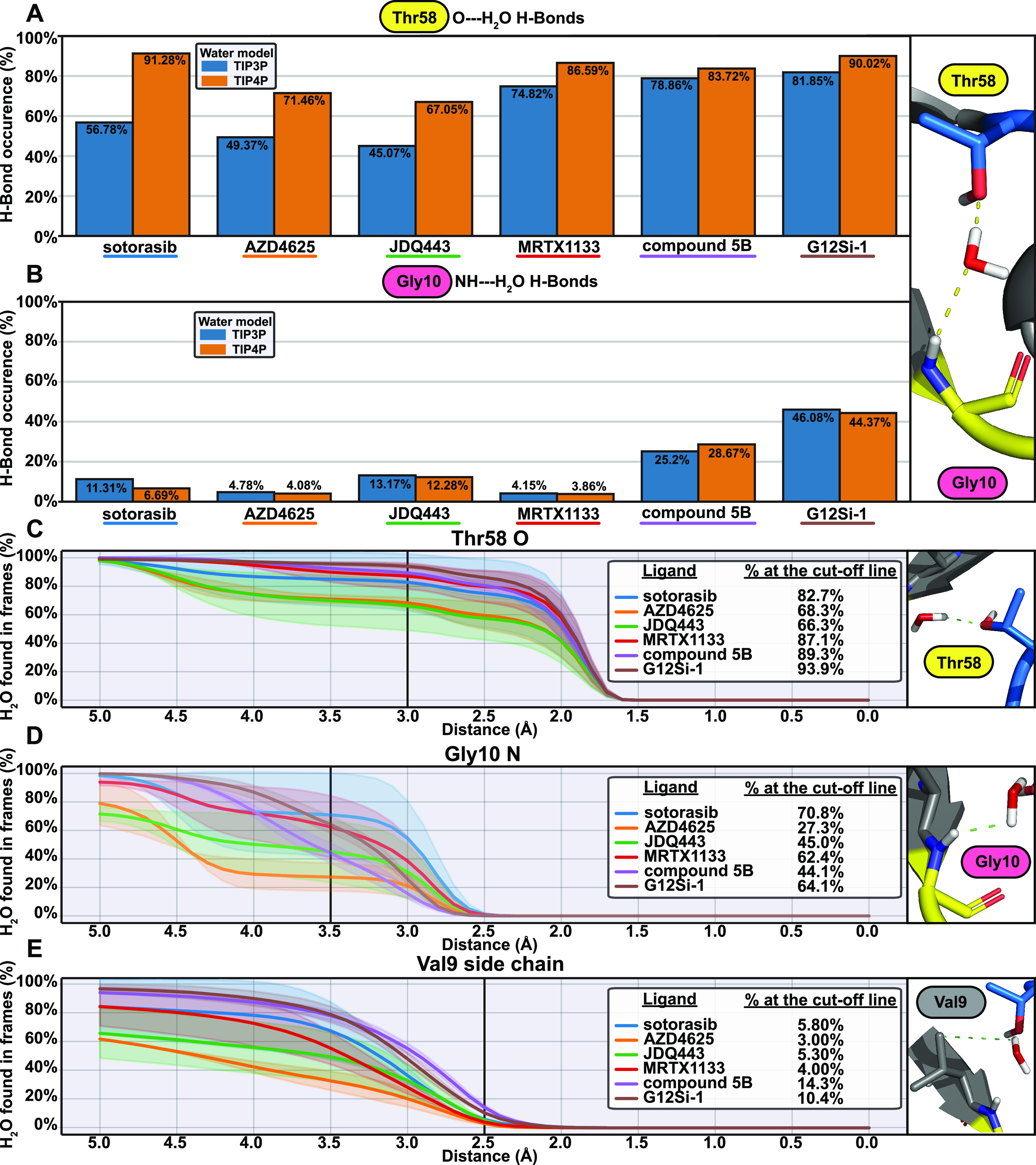
Water behavior on the conserved water location in microsecond timescale MD simulations. (A) Observed H-bond frequencies between Thr58 O and water in simulated systems. (B) Observed H-bond frequencies between Gly10 NH and water in simulated systems. (C) Minimum distance of a water molecule to the oxygen (O) atom of Thr58. (D) Minimum distance of a water molecule to the nitrogen (N) of Gly10. (E) Minimum distance of a water molecule to the side chain atoms of Val9. In (C)–(E), the line displays the average, and the shaded area represents SD.

Next, we examined the conserved water behavior in the simulations by monitoring the water H-bonds and distances to Thr58 and Gly10 (Figure 9). The observed H-bond frequencies between the side chain oxygen of Thr58 and water appear to depend on the water model. The TIP4P water model displays much higher frequencies for Thr58–water H-bonds compared to TIP3P, and this difference occurs with higher magnitude with the compounds that possess the highest WaterMap-predicted energies (**sotorasib**, **AZD4625**, **JDQ443**, and **MRTX1133**). The discrepancy between the water models in Gly10(NH)–water H-bonds is less profound. The lowest frequencies of Gly10(NH)–water H-bonds are observed for **AZD4625** and **MRTX1133**, while compounds **5B** and **G12Si-1** exhibit the highest frequencies. Water presence near Thr58 oxygen is the lowest with **AZD4625** and **JDQ443** based on the minimum water distance (Figure 9C), while **AZD4625** also appears to push the water away from Gly10 nitrogen (Figure 9D). Generally, we observed lower distances between water and the investigated protein atom(s) with the TIP4P water model compared to TIP3P (Figure S19). The increased frequency of Thr58 H-bonds with TIP4P may be associated with differences in the partial charges of the water models (H: +0.520 TIP4P and +0.417 TIP3P).

Finally, we analyzed the putative additional water presence in the simulations near the conserved water side on the back of the SII-P, where the side chain of Val9 is located. The minimum water distance analysis suggests an increased preference for water in this location with compounds **5B** and **G12Si-1**, compared to other systems (Figure 9E). Indeed, WaterMap predicted an adjacent hydration site for compound **5B** (Table 2, Figure S9), but the same was not observed for **G12Si-1** (Figure 5A). The water existence (and the vacant space for it) near Val9 could be related to the fact that the **G12Si-1** is less stable on the binding site compared to other compounds (Figures S15, S17, and S18).

## Discussion

Here, we analyzed 70 publicly available KRAS SII-P binder cocrystal structures related to Thr58-associated conserved water and found that it is present in most of the KRAS SII-P binder cocrystal structures (apart from a small subset of the G12C inhibitors and the first-reported G12R inhibitor). This conserved water likely plays an important role in switch-II functionality, as it connects β1 sheet (Gly10) to the highly flexible switch-II (Thr58), and it is observed with numerous KRAS structures, including active signaling state structures (KRAS–effector protein complexes) (Table S1). KRAS T58I mutations have been found in Noonan syndrome,^56^ and it also appears rarely in different cancers (14 patients in GENIE Cohort v.12.0-public).^57,58^ The biochemical consequences of T58I are still unclear but most likely KRAS-activating.^59^ Overall, this highlights the importance of the key Thr58 residue in KRAS functionality.

The initial KRAS(G12C) inhibitors reported by Ostrem et al.^34^ display more ambiguous characteristics with the conserved water (and the switch-II region), while the later more potent inhibitors are often accompanied by a single isolated high-energy hydration site. Only a subset of covalent inhibitors is capable of displacing the Thr58-associated conserved water. One of these inhibitors, **adagrasib**, was shown to bind WT KRAS with higher affinity than **sotorasib**.^60^ We noted here that the disclosed water-replacing compounds are somewhat suboptimal, as, for instance, a cyanomethyl group as the water displacer appears to introduce a repulsion with the native conformation of Thr58 (manifested with the Thr58 side chain conformation change). To date, most of the reported SII-P binders are covalent inhibitors targeting KRAS(G12C) (and more recently G12S and G12R). Therefore, it may be that maintaining the inhibitor reactivity to the covalent linkage to position 12, located near this conserved water region, has hindered the water-targeting among these compounds. Obviously, after the covalent reaction has taken place, the consideration of the initial negative water impact on compound efficacy is less profound.

With the noncovalent Mirati series targeting KRAS(G12D), the cyanomethyl water-displacing approach proved unbeneficial.^6^ Overall, no noncovalent binders that are capable of displacing the conserved water have been reported to date. Nevertheless, based on our results, Mirati’s most potent KRAS(G12D) binders displace the adjacent high-energy water molecules of the Thr58-associated hydration site, and a similar trend is observed with the G12C inhibitors. Therefore, as it seems beneficial to displace at least the adjacent hydration sites next to the conserved water site, we speculate that the shielding of this water (if it is not displaced) is a necessity for efficient SII-P binding.

The static crystal structure represents only one conformation (or state) of the protein. As WaterMap is highly sensitive to the input coordinates, this may have an impact on these results. Also, the crystal structures are usually resolved at cryogenic temperatures, which has been recently demonstrated to have an effect on the water networks, potentially resulting in different water conformation preferences from those at room temperature (or in the physiological temperature).^61^ The crystal structure data related to water should always be interpreted with some caution considering these findings. The high energy of the conserved water with the SII-P binder in this KRAS conformation may offer a partial explanation of the observed high fluctuation of switch-II in microsecond timescale molecular dynamics simulations as observed with the inhibitors here and earlier with **sotorasib**.^62^ Our results suggest that the inhibitors display discrepancy in their impact on the conserved water behavior and its interactions with KRAS. Among the studied inhibitors, **JDQ443** and **AZD4625** appeared the most repulsive for the conserved water, while **G12Si-1** and compound **5B** appeared the least. Obviously, this discrepancy may also be partially mediated via the inhibitor–switch-II interactions, as switch-II fluctuations and conformation are interlinked to the water access to this conserved site. Interestingly, a dramatic instability of the ligand near the conserved water site was observed in one of the simulation replicas of compound **5B**. This compound was also the only one from the simulated set for which WaterMap predicted an adjacent hydration site next to the conserved water site. Related to the used water models on the conserved water site, the TIP4P water model exhibited closer contacts to the protein and more stable interactions, while TIP3P was more dynamic and reflected better the WaterMap results.

Overall, our results highlight the high-energy characteristics of this Thr58-associated conserved water when coexisting with an SII-P binder. Therefore, there resides great potential in targeting this water molecule. However, this is not a trivial task as the pocket is highly dynamic,^15^ and it will require an optimally designed compound that is capable of replacing the water without simultaneously compromising the binding affinity of the small molecule in other regions. To achieve this goal, tremendous efforts from medicinal chemists and other scientists are required. Finally, we emphasize that one should not have any unrealistic expectations related to conserved water, it will not solely define the binding affinity of a compound, and it is only one piece of a much larger puzzle.

## Materials and Methods

Molecular modeling was conducted with Maestro (Schrödinger Release 2021-4, Schrödinger LLC, New York, NY, 2021) and OPLS4 force field.^63^ All publicly available KRAS structures with SII-P binders were downloaded from RCSB Protein Data Bank and prepared and energy-minimized using Protein Preparation Wizard,^64^ adding the missing side chains and loops with Prime^65,66^ and keeping the crystal waters within 5 Å of the ligands (deleting the other waters). We deleted closely residing cocrystallized excipients near the SII-P from the structures, which would have potentially impacted WaterMap results. We excluded the two structures from the SII-P binder analysis (PDB IDs: 7U8H and 8AFD) that contain SII-P-binding fragments that are cocrystallized with a secondary site covalent small molecule and with a different position 12 mutant (KRAS(G12V, S39C-BIT)+GDP).^52^

WaterMap^30,31^ simulations were conducted with default settings, including waters to the analysis within 10.0 Å range of the SII-P-bound ligand. For the structures without the SII-P binder, residues Thr58, Met72, and Tyr96 were selected to define the site. The water molecules near the ligand (originating from the crystal structure) were treated as solvent. A simulation time of 2.0 ns was applied. For all systems, if not otherwise stated, we used chain A for the WaterMap simulations (except for 6B0Y the WaterMap was run for chain B only).

The Desmond^67^ molecular dynamics engine was used for the simulations of the selected systems. The prepared protein structures (PDB IDs: 6OIM, 7R0M, 7O70, 7RPZ, 7RT4, 7TLE) were solvated in a cubic box with a minimum distance of 15 Å from the protein or the ligand to the edges, with K^+^ and Cl^-^ ions, to neutralize the system, and with 150 mM concentration. For each system, two different water models, TIP3P and TIP4P,^33^ were applied. Desmond simulations were run in the NpT ensemble at 1.01325 bar and 300 K (Martyna–Tobias–Klein barostat and Nosé–Hoover chain thermostat) with the default settings of RESPA integrator timesteps with 2, 2, and 6 fs applied for bonded, near, and far, respectively. The default Coulombic cutoff of 9 Å was applied. Before the production simulations, the default Desmond system relaxation protocol was applied. Each system was simulated for 1000 ns with 10 replicas using a random seed, resulting in a total of 120 μs aggregate of simulation data.

Protein–ligand interactions, protein RMSF, and ligand RMSD were analyzed by the Simulation Interaction Analysis tool (scripts: event_analysis.py; analyze_simulation.py) (Schrödinger LLC). The default settings were used in the definition of the interactions, where the following parameters were applied: H-bonds, a distance of 2.5 Å between the donor and acceptor with ≥120 and ≥90° for donor and acceptor angles, respectively; π–cation interactions, a 4.5 Å distance between the positively charged and aromatic group; π–π interactions, stacking of two aromatic groups face-to-face or face-to-edge; water bridges, a distance of 2.8 Å between the donor and acceptor with ≥110 and ≥90° for donor and acceptor angles, respectively. The minimum distances between the selected atom(s) and water were monitored by the trajectory_asl_monitor.py script (Schrödinger LLC). For selected analysis, replica trajectories were merged before the analysis using the trj_merge.py script (Schrödinger LLC). The output was transformed to the csv format by st2csv.py (Schrödinger LLC) and plotted by seaborn (v0.11.2).^68^

Visualization of the electron density data was conducted with Protein Data Bank in Europe’s (PDBe) 3D visualizer Mol*.^69,70^ WaterMap illustrations were made with PyMOL (The PyMOL Molecular Graphics System, Version 2.5.0 Schrödinger, LLC).

## Data Availability

WaterMap results are freely available at: https://doi.org/10.5281/zenodo.7656467. Raw trajectories of the Desmond molecular dynamics simulations are freely available at: https://doi.org/10.5281/zenodo.7341954 (TIP3P) and https://doi.org/10.5281/zenodo.7342311 (TIP4P).

## Supporting Information Available

Additional images of the WaterMap results, crystal structure electron density maps, and MD simulation results (Figures S1–S19) (PDF)
Publicly available X-ray structures of KRAS and the presence of Thr58-associated conserved water (Table S1) (XLSX)
2D structures of the SII-P binders with publicly available structures (Table S2) (PDF)
WaterMap data of the conserved hydration site provided in the tables in more detail (XLSX)
SMILES are provided for all of the compounds reported in the manuscript (XLSX)

## Abbreviations

SII-P: switch-II pocket
MD: molecular dynamics
GDP: guanosine diphosphate
GNP: phosphoaminophosphonic acid guanylate ester
GTP: guanosine triphosphate
GCP: phosphomethylphosphonic acid guanylate ester
