# Supplementary material for: In Silico Evaluation of the Thr58-Associated Conserved Water with KRAS Switch-II Pocket Binders
Source: J Chem Inf Model. 2023 Feb 28;63(5):1490–505. doi: 10.1021/acs.jcim.2c01479 (PMC10015465; doi:10.1021/acs.jcim.2c01479)
Supplement: Supplementary file 1 — ci2c01479_si_001.pdf [file ci2c01479_si_001.pdf]

Supporting Information

In silico Evaluation of the Thr58-associated Conserved Water with KRAS Switch-II Pocket Binders

*Renne Leini[1], Tatu Pantsar[1]**

[1]School of Pharmacy, Faculty of Health Sciences, University of Eastern Finland, Yliopistoranta 1C, 70210 Kuopio, Finland

*Email: tatu.pantsar@uef.fi

KEYWORDS. KRAS; water; WaterMap; molecular dynamics simulations; drug design.

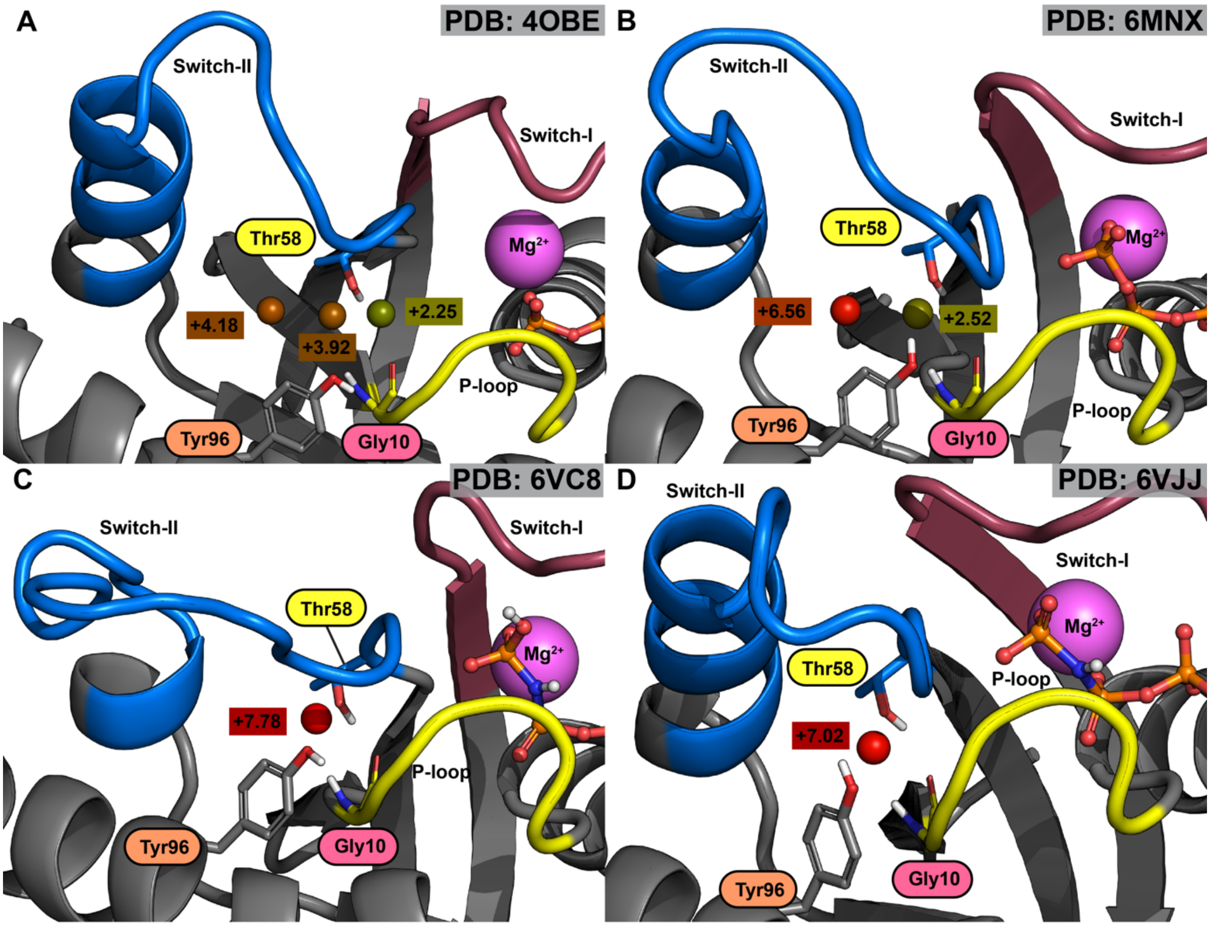

Figure S1. WaterMap results of selected KRAS structures without SII-P binders (PDB IDs: 4OBE, 6MNX, 6VC8 and 6VJJ). (**A**) Two adjacent hydration sites (ΔG = +4.18 and +2.25 kcal/mol) appear next to the conserved site (ΔG = +3.92 kcal/mol) in PDB ID: 4OBE. (**B**) One adjacent hydration site (ΔG = +6.56 kcal/mol) exists next to the conserved site (ΔG = +2.52 kcal/mol) in PDB ID: 6MNX. (**C**) An isolated conserved hydration site (ΔG = +7.78) is observed in PDB ID: 6VC8. (**D**) An isolated conserved hydration site (ΔG = +7.02) is observed in PDB ID: 6VJJ.

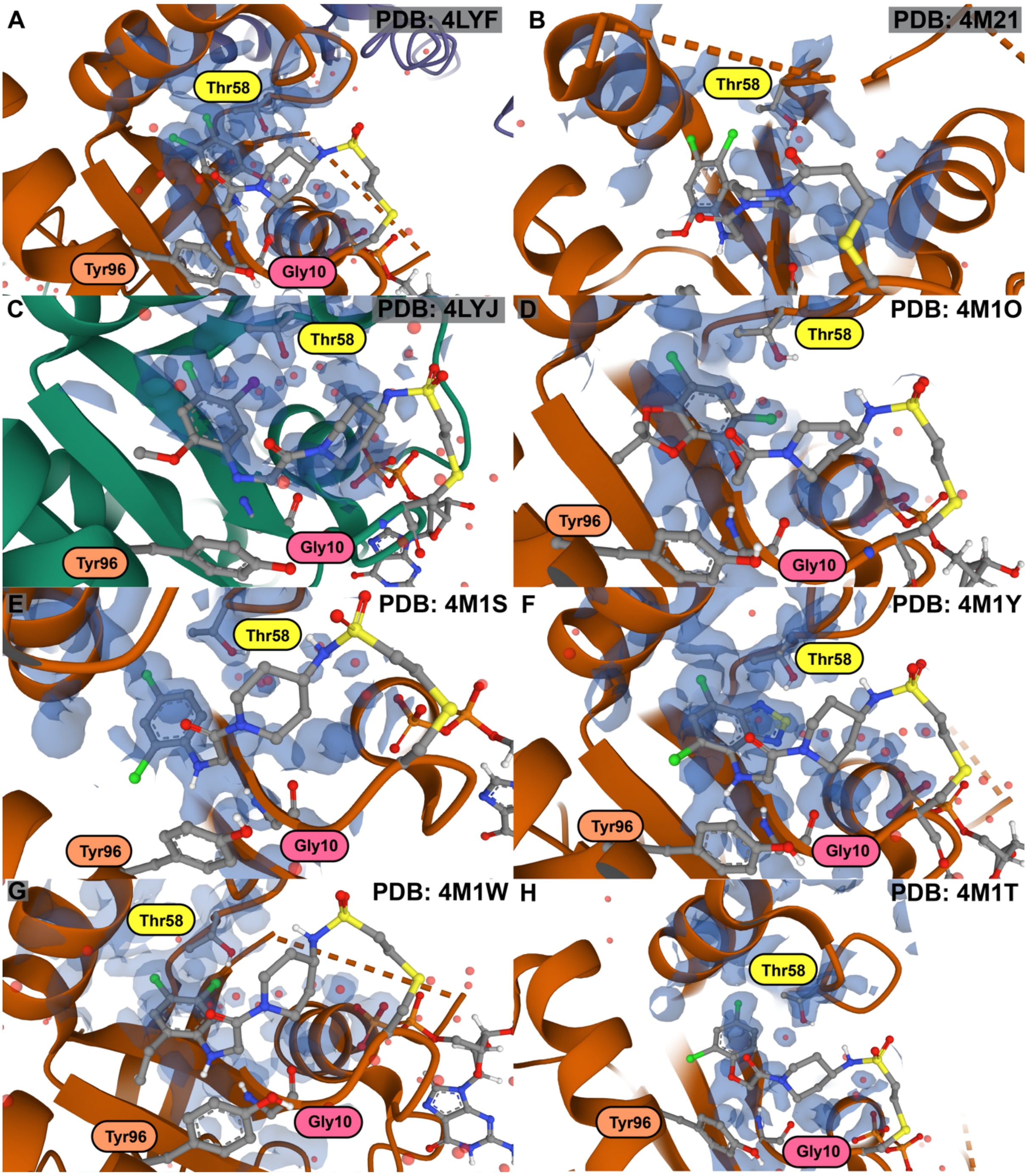

Figure S2. Different switch-II configuration containing KRAS structures with SII-P binders. Switch-II appears in different more open conformation with Thr58 found in different location in multiple early G12C binders reported by Ostrem *et al*.: (**A**) "Compound **8**" (PDB ID: 4LYF), (**B**) Switch-II is disordered with "Compound **11**" (PDB ID: 4M21), (**C**) "Compound **9**" (PDB ID: 4LYJ), (**D**) "Compound **7**" (PDB ID: 4M1O), (**E**) "Compound **13**" (PDB ID: 4M1S), (**F**) "Compound **15**" (PDB ID: 4M1Y), (**G**) Unnamed ligand (PDB ID: 4M1W), (**H**) "Compound **14**" (PDB ID: 4M1T). Electron density (blue transparent surface) displayed at 2Fo-Fc σ = 1.5.

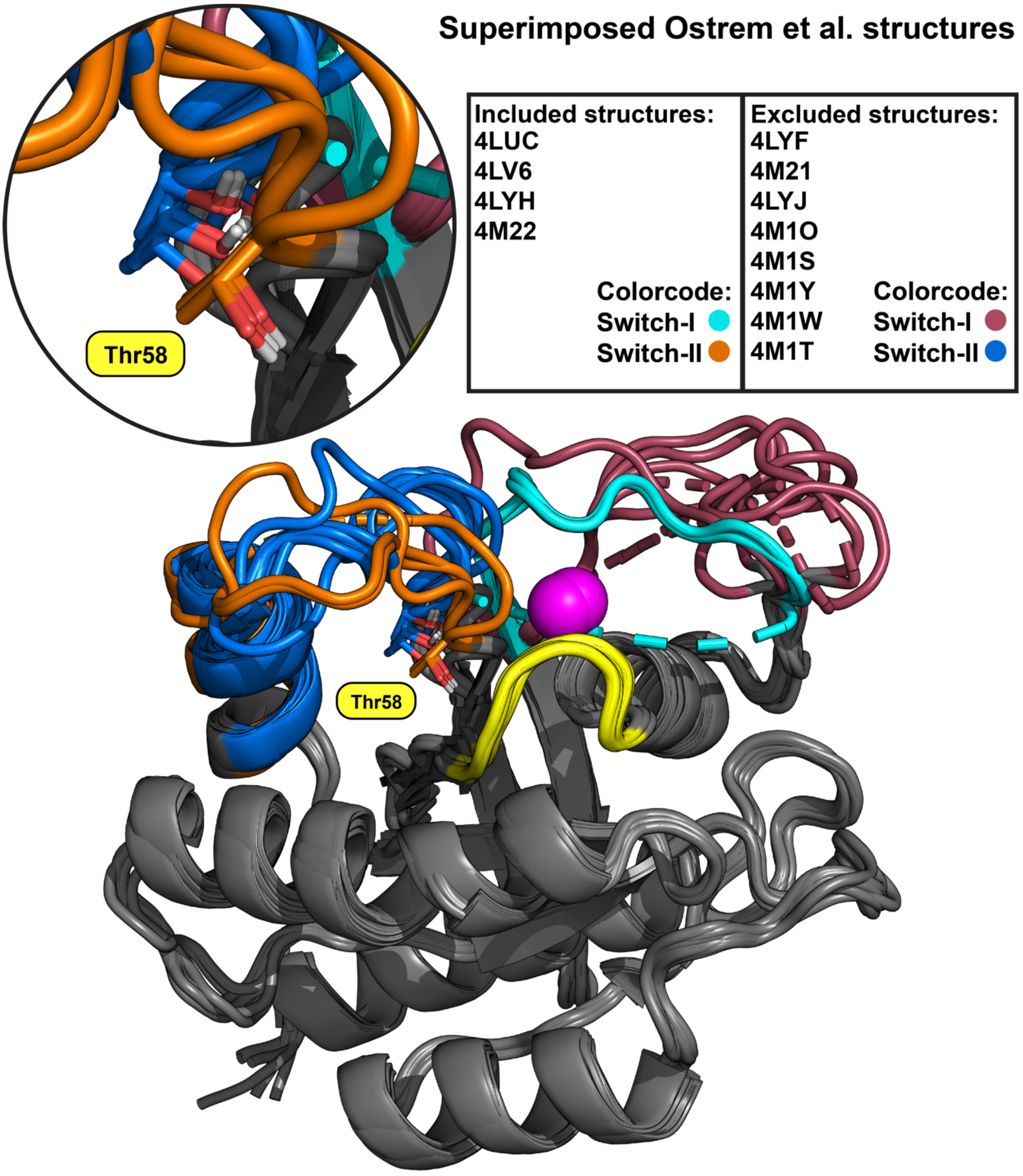

Figure S3. Superimposed Ostrem et al. structures. The excluded structures appear in a distinct Thr58 configuration, while the included structures appear in the specific Thr58 orientation that is found in numerous KRAS structures.

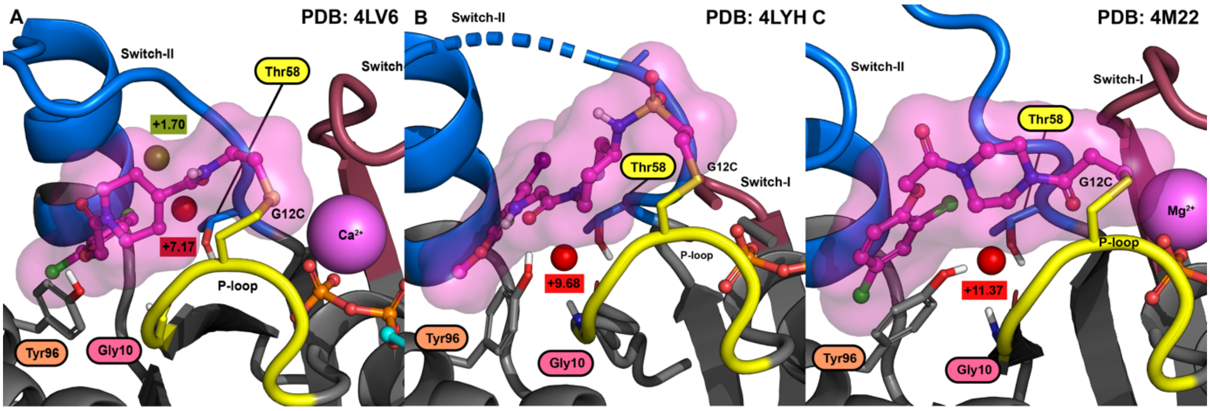

Figure S4. WaterMap results of PDB IDs: 4LV6, 4LYH and 4M22. (**A**) "Compound **4**" displays one adjacent hydration site ($\Delta G = +1.70$ kcal/mol) next to the conserved site ($\Delta G = +7.17$ kcal/mol) (PDB ID: 4LV6). (**B**) "Compound **9**" displays an isolated conserved hydration site ($\Delta G = +9.68$ kcal/mol) (PDB ID: 4LYH). (**C**) "Compound **16**" displays an isolated conserved hydration site ($\Delta G = +11.37$ kcal/mol) (PDB ID: 4M22).

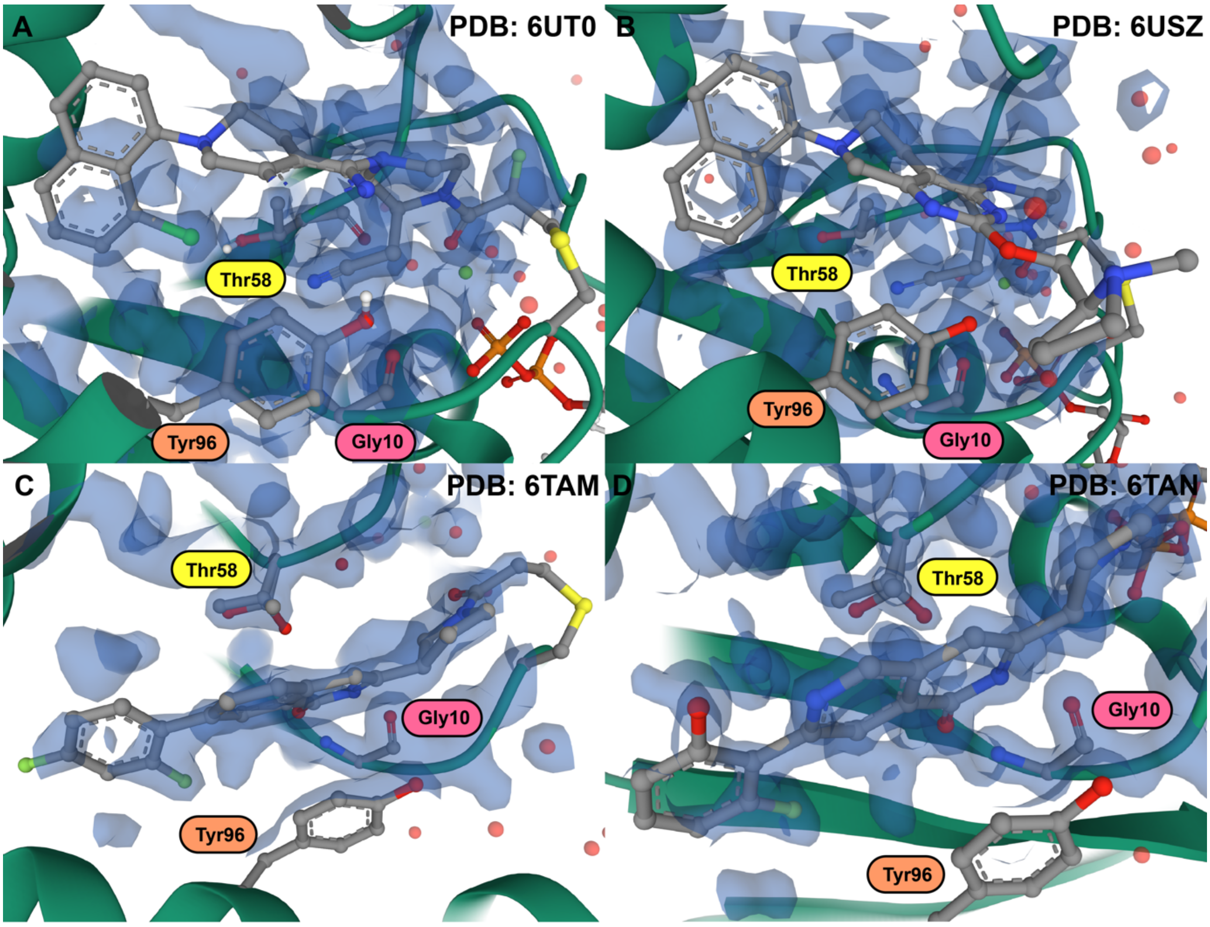

Figure S5. Electron density of Thr58 and its conformation probabilities with the structures inducing its conformation change. (**A**) Adagrasib (PDB ID: 6UT0) and (**B**) "compound **12a**" (PDB ID: 6USZ) exhibit solely flipped configuration of Thr58, while normal and flipped configuration of Thr58 are both observed with 0.5 probabilities for (**C**) "compound **3**" (PDB ID: 6TAM) and (**D**) "compound **13**" (PDB ID: 6TAN). Electron density (blue transparent surface) displayed at 2Fo-Fc $\sigma = 1.5$.

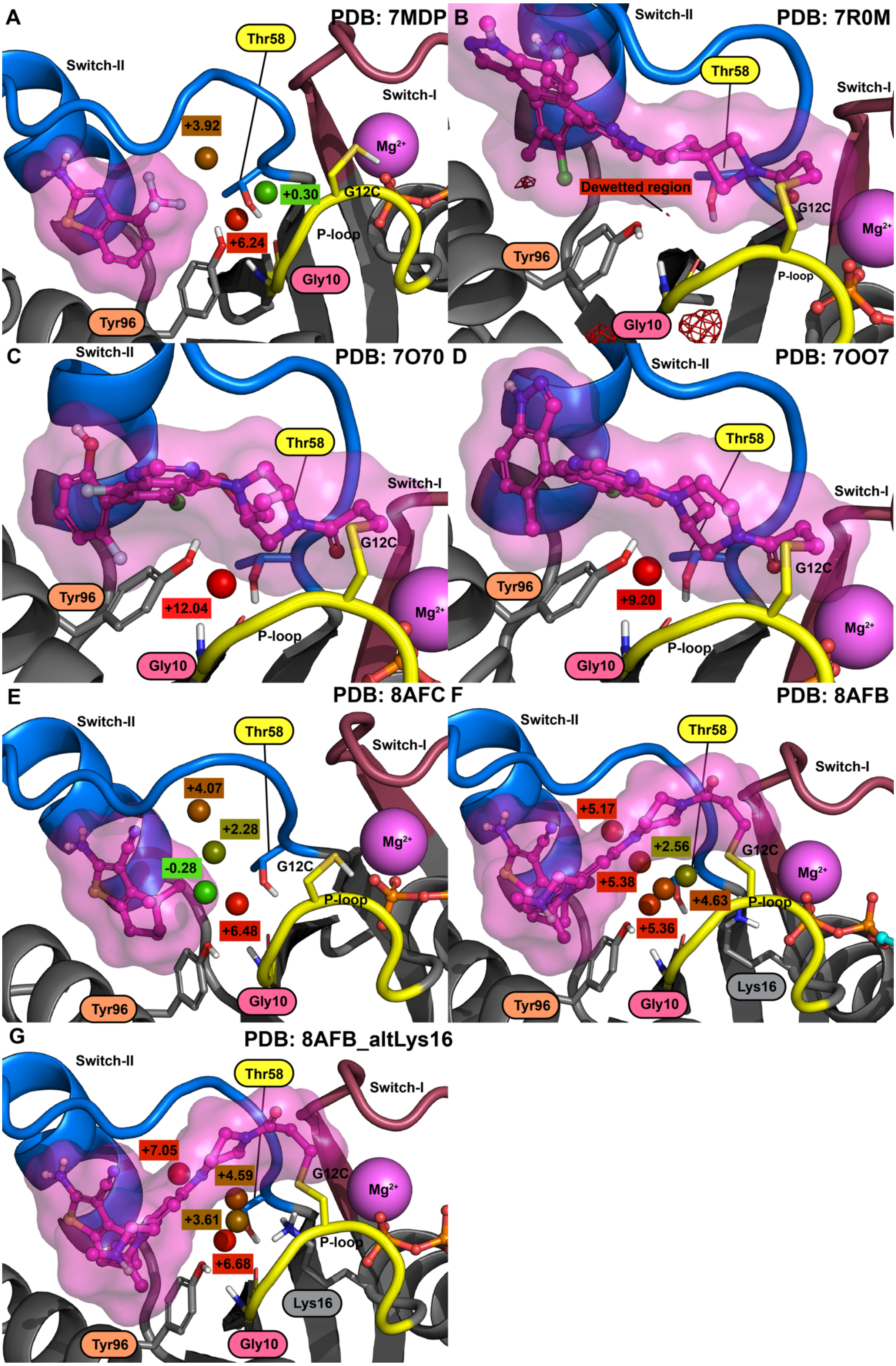

A
PDB: 7MDP
Switch-II
Thr58
Switch-I
+3.92
Mg2+
+0.30
G12C
+6.24
P-loop
Gly10
Tyr96
B
PDB: 7R0M
Switch-II
Thr58
Switch-I
Dewetted region
G12C
Mg2+
Tyr96
P-loop
Gly10
C
PDB: 7O70
Switch-II
Thr58
Switch-I
G12C
Tyr96
+12.04
Mg2+
P-loop
Gly10
D
PDB: 7OO7
Switch-II
Thr58
Switch-I
Tyr96
G12C
+9.20
Mg2+
P-loop
Gly10
E
PDB: 8AFC
Switch-II
Thr58
Switch-I
+4.07
+2.28
Mg2+
G12C
-0.28
P-loop
+6.48
Gly10
Tyr96
F
PDB: 8AFB
Switch-II
Thr58
Switch-I
+5.17
+2.56
G12C
Mg2+
+5.38
+4.63
P-loop
+5.36
Lys16
Tyr96
Gly10
G
PDB: 8AFB_altLys16
Switch-II
Thr58
Switch-I
+7.05
+4.59
G12C
Mg2+
+3.61
P-loop
+6.68
Lys16
Tyr96
Gly10

Figure S6. WaterMap results of PDB IDs: 7MDP, 7R0M, 7O70, 7OO7, 8AFC and 8AFB. (**A**) **GNE-2897** displays adjacent hydration sites (ΔG = +3.92 and +0.30 kcal/mol) next to the conserved site (ΔG = +6.24 kcal/mol) (PDB ID: 7MDP). (**B**) **JDQ443** exhibits a high-energy de-wetted region on the conserved water site (PDB ID: 7R0M). (**C**) **AZD4625** displays an isolated conserved hydration site (ΔG = +12.04 kcal/mol) (PDB ID: 7O70). (**D**) "Compound **28**" displays an isolated conserved hydration site (ΔG = +9.20 kcal/mol) (PDB ID: 7OO7). (**E**) "Compound **12**" displays adjacent hydration sites (ΔG = +2.28, +4.07 and -0.28 kcal/mol) next to the conserved site (ΔG = +6.48 kcal/mol) (PDB ID: 8AFC). (**F**) **BI-0474** displays adjacent hydration sites (ΔG = +4.63, +5.38, +2.56 and +5.17 kcal/mol) next to the conserved site (ΔG = +5.36 kcal/mol) (PDB ID: 8AFB). (**G**) **BI-0474** with its alternative Lys16 configuration displays adjacent hydration sites (ΔG = +3.61, +4.59 and +7.05 kcal/mol) next to the conserved site (ΔG = +6.68 kcal/mol) (PDB ID: 8AFB).

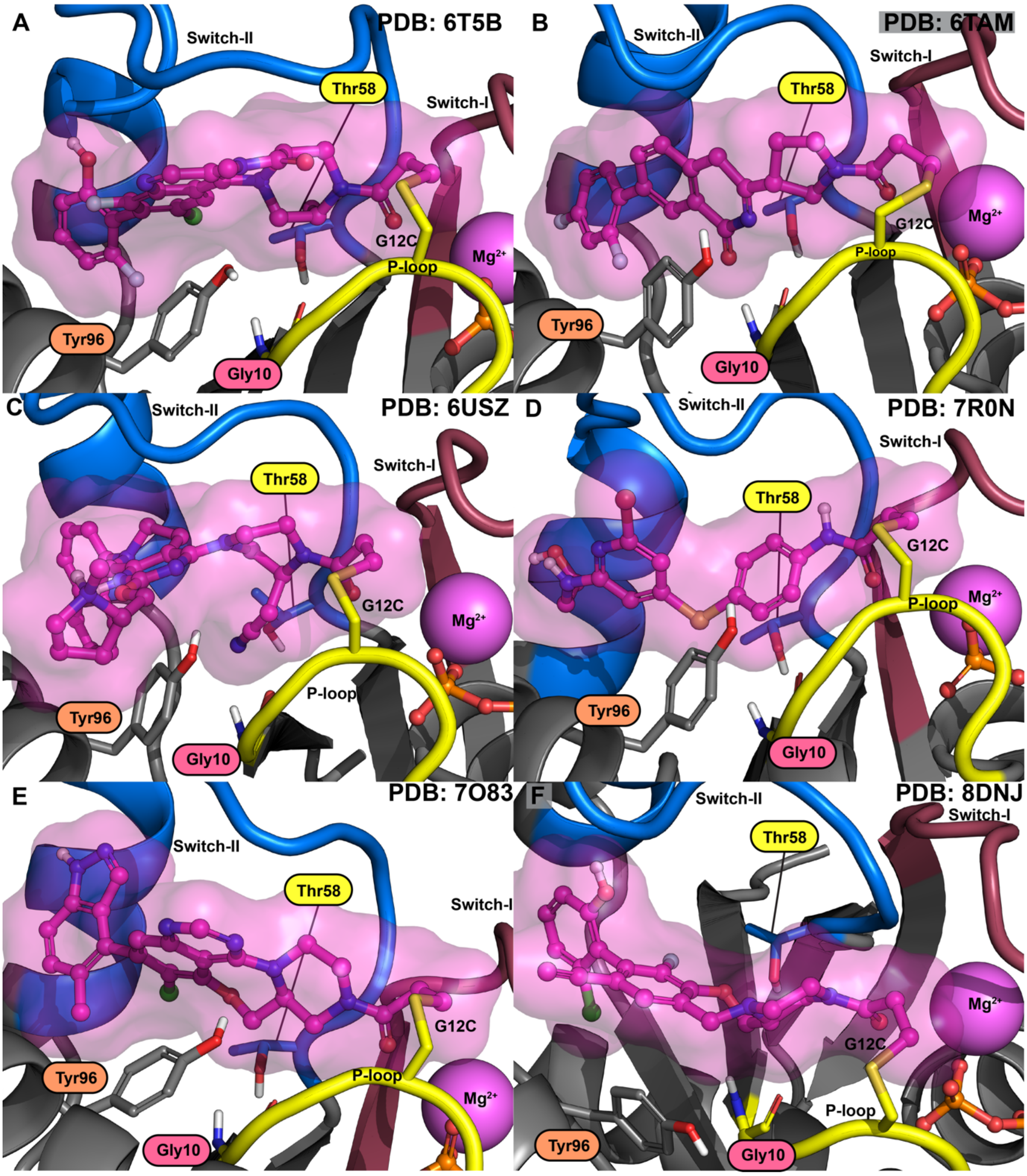

Figure S7. WaterMap results of PDB IDs: 6TB5, 6TAM, 6USZ, 7R0N, 7O83 and 8DNJ. (**A**) "Compound **25**" displaces conserved water with methyl group (PDB ID: 6TB5). (**B**) "Compound **3**" displaces conserved water with its *iso*quinolinone-moiety (PDB ID: 6TAM). (**C**) "Compound **12a**" displaces conserved water with cyanoethyl-moiety (PDB ID: 6USZ). (**D**) "Compound **2**" displaces conserved water with a phenyl ring (PDB ID: 7R0N). (**E**) "Compound **23**" displaces conserved water with a distal methyl in its heterocyclic ring system (PDB ID: 7O83). (**F**) **WO2020/178282A1** displaces conserved water with a distal methyl in its heterocyclic ring system (PDB ID: 8DNJ).

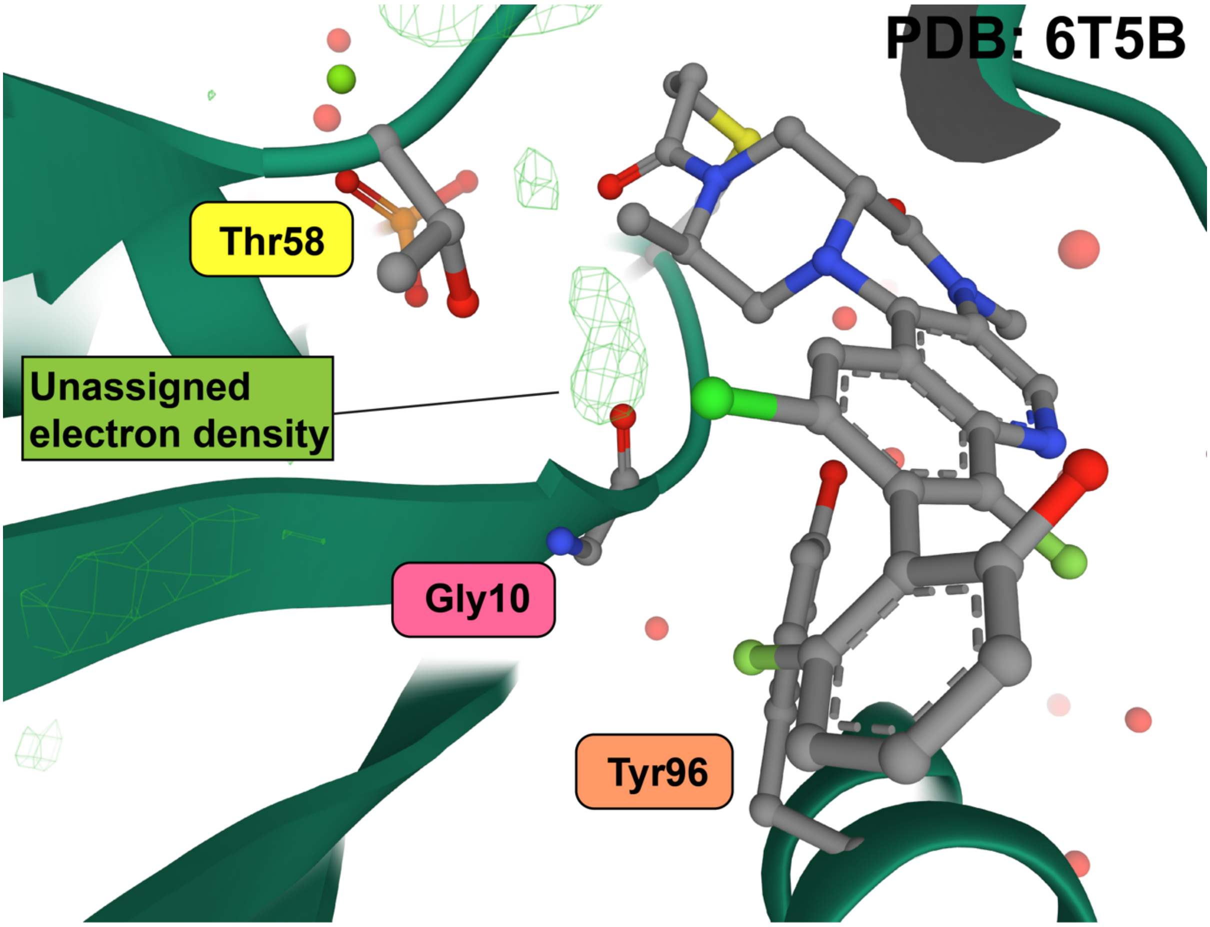

Figure S8. Unassigned electron density on the conserved water site of the 6T5B structure. Positive electron density is observed on the conserved water site with "Compound **25**" (PDB ID: 6T5B) according to the Fo-Fc(+ve) σ = 3 map, which is illustrated in green mesh.

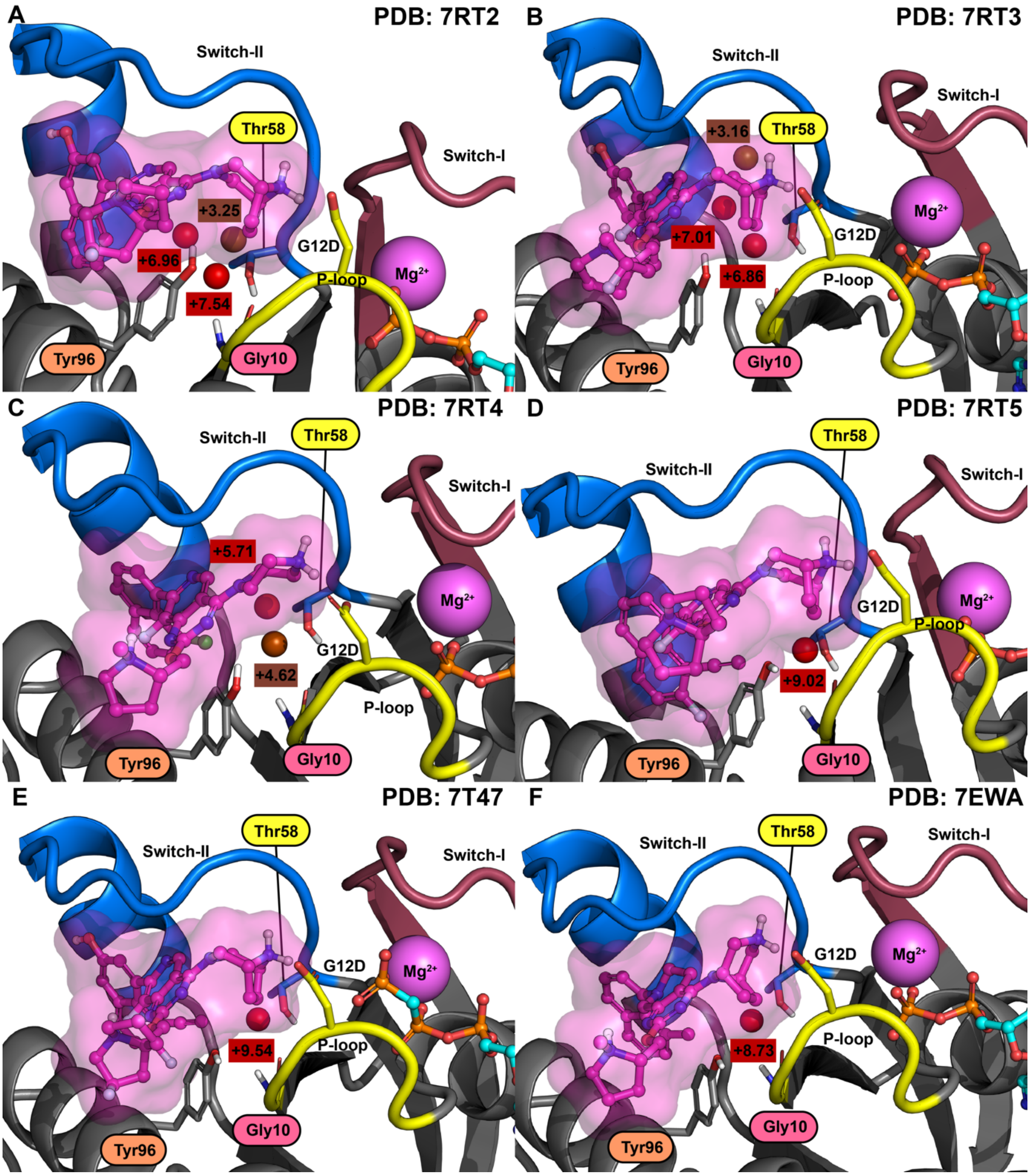

Figure S9. WaterMap results of PDB IDs: 7RT2, 7RT3, 7RT4, 7RT5, 7T47 and 7EWA. (**A**) "Compound **25**" displays adjacent hydration sites (ΔG = +6.96 and +3.25 kcal/mol) next to the conserved site (ΔG = +7.54 kcal/mol) (PDB ID: 7RT2). (**B**) "Compound **24**" displays adjacent hydration sites (ΔG = +7.01 and +3.16 kcal/mol) next to the conserved site (ΔG = +6.86 kcal/mol) (PDB ID: 7RT3). (**C**) "Compound **5B**" displays an adjacent hydration site (ΔG = +5.71 kcal/mol) next to the conserved site (ΔG = +4.62 kcal/mol) (PDB ID: 7RT4). (**D**) "Compound **36**" displays an isolated conserved hydration site (ΔG = +9.02 kcal/mol) (PDB ID: 7RT5). (**E**) **MRTX1133** displays an isolated conserved hydration site (ΔG = +9.54 kcal/mol) also when in complex with GCP bound KRAS (PDB ID: 7T47). (**F**) **TH-Z827** displays an isolated conserved hydration site (ΔG = +8.73 kcal/mol) (PDB ID: 7EWA).

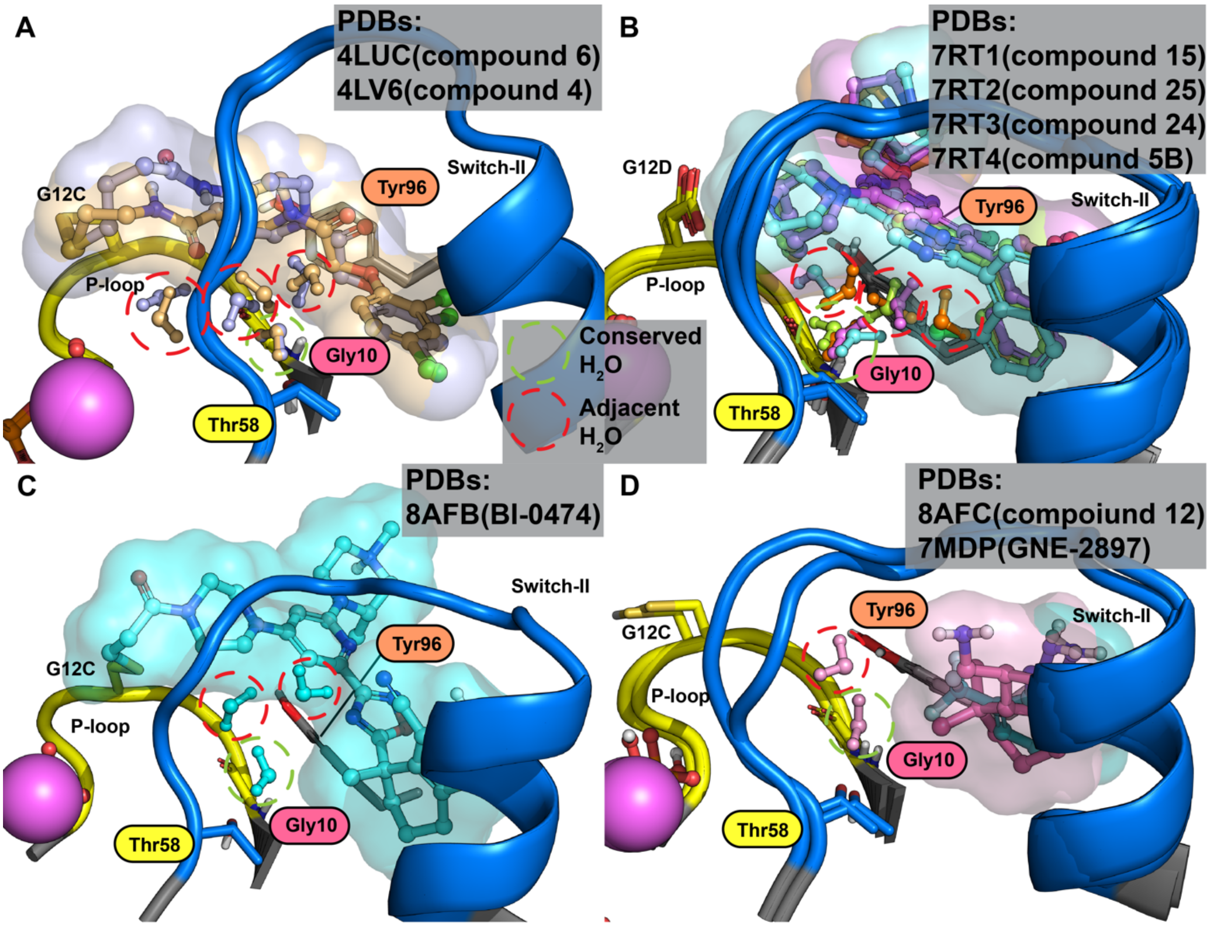

Figure S10. Structures with WaterMap predicted adjacent hydration sites display adjacent water molecules in their crystal structures. The superimposed structures are grouped into **A–D** based on their SII-P binder scaffolds. The only exception here, without any assigned waters in the site is 7MDP (**D**).

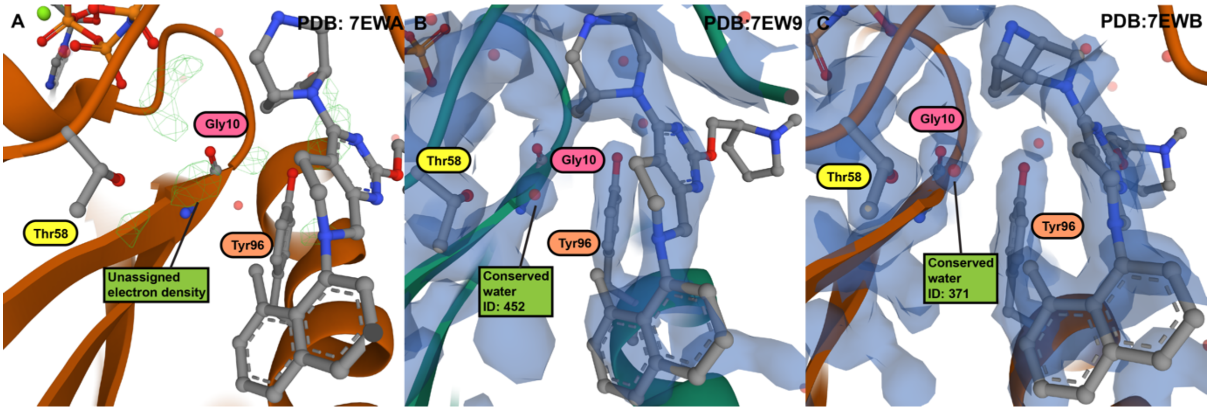

Figure S11. Unassigned electron density at the conserved water location indicates the presence of water in the structure PDB ID: 7EWA. (**A**) Positive electron density is observed on the conserved water site with **TH-Z827** (PDB ID: 7EWA) according to the Fo-Fc(+ve) $\sigma = 3$ map, which is illustrated in green mesh. (**B**) Conserved water is present in the structure of **TH-Z816** (PDB ID: 7EW9). (**C**) Conserved water is present in the structure of **TH-Z835** (PDB ID: 7EWB). Electron density (blue transparent surface) displayed at 2Fo-Fc $\sigma = 1.5$.

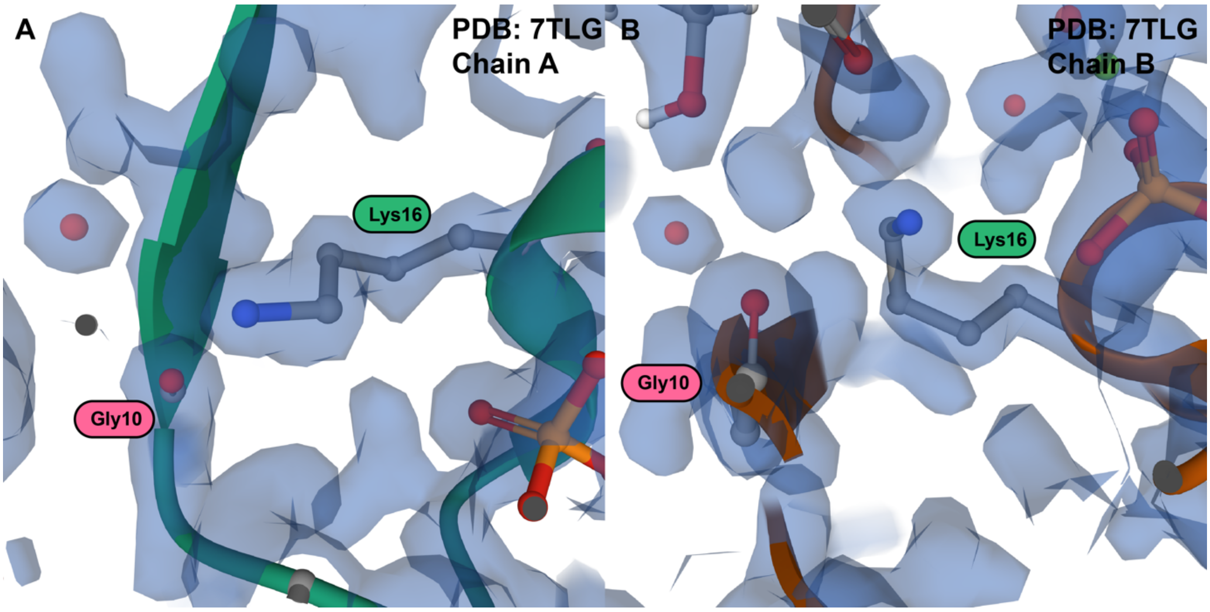

Figure S12. Discrepancy of Lys16 conformation on chain A and B in the 7TLG structure. (**A**) In chain A of **G12Si-5** structure (PDB ID: 7TLG), Lys16 is observed in a shifted conformation where it is oriented towards the SII-P. (**B**) In chain B, Lys16 appears in its normal configuration, where it is observed in most KRAS structures. Electron density (blue transparent surface) displayed at 2Fo-Fc $\sigma = 1.5$.

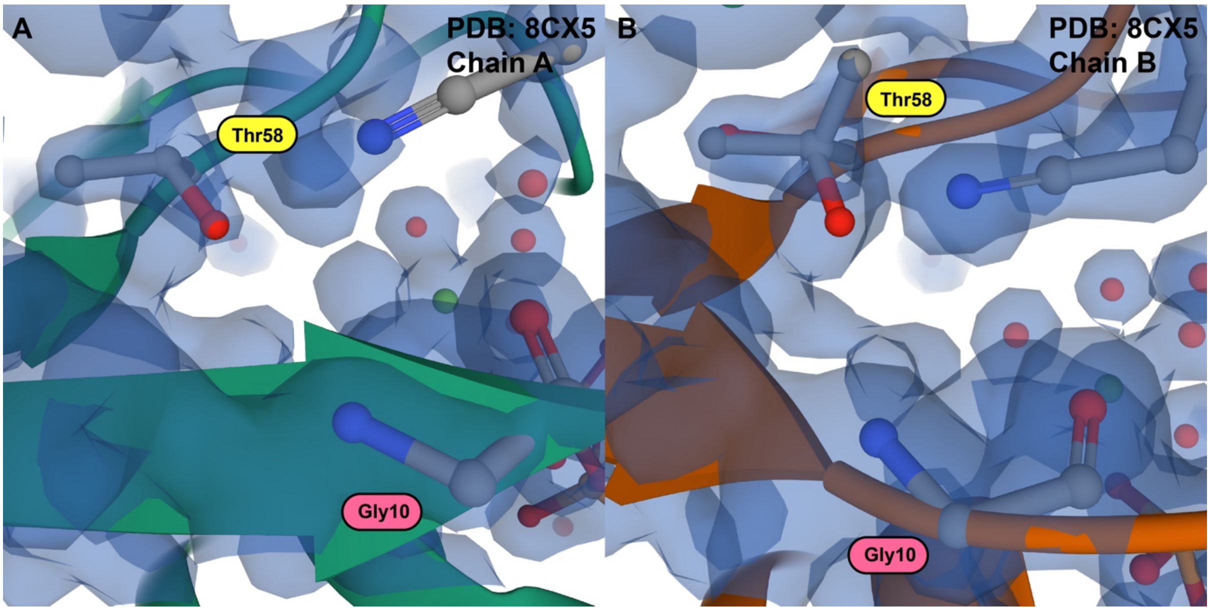

Figure S13. Electron density of Thr58 and its conformation probabilities in the structure PDB ID: 8CX5. (**A**) In chain A of "Compound **4**" (PDB ID: 8CX5) Thr58 is observed solely in normal conformation, while in chain B (**B**) normal and flipped configuration of Thr58 are observed with 0.29 and 0.71 probabilities, respectively. Electron density (blue transparent surface) displayed at 2Fo-Fc σ = 1.5.

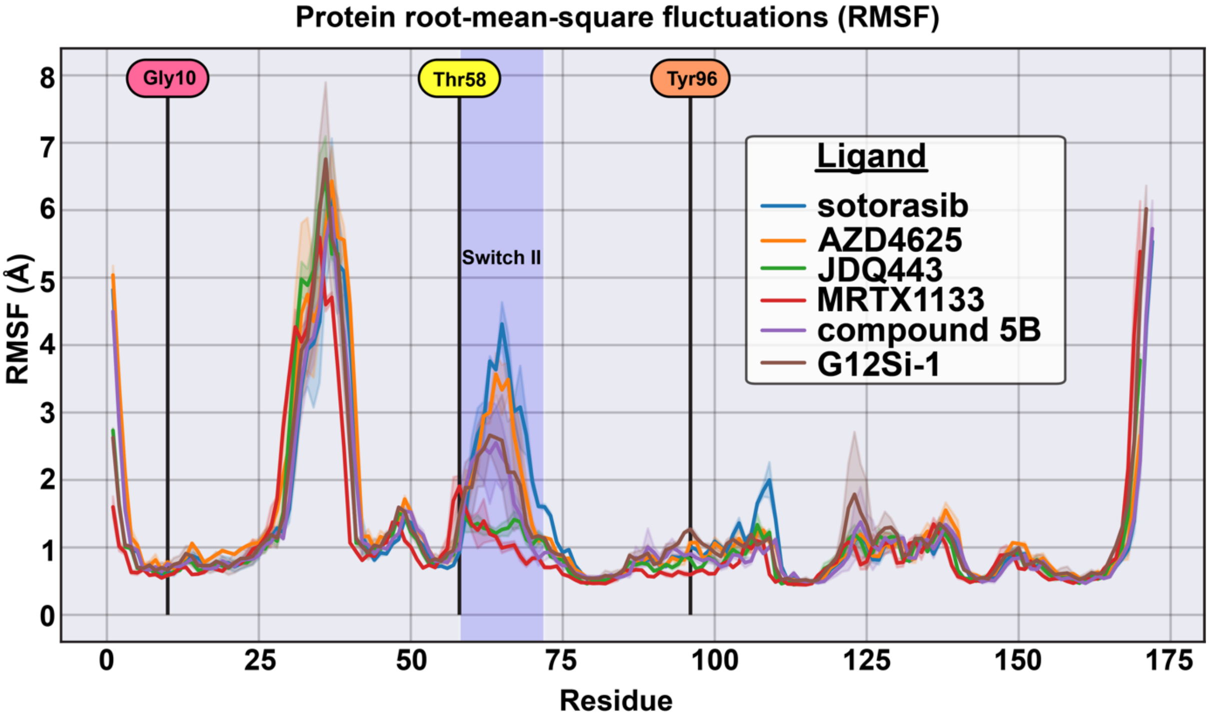

Figure S14. Protein root-mean-square fluctuations (RMSF) in microsecond timescale MD simulations. Average RMSF values of each system (combined TIP3P and TIP4P simulations) is shown with line with SD highlighted with shaded area. Switch-II region (residues 58–72) is highlighted with blue. Data consist of 20 µs for each ligand.

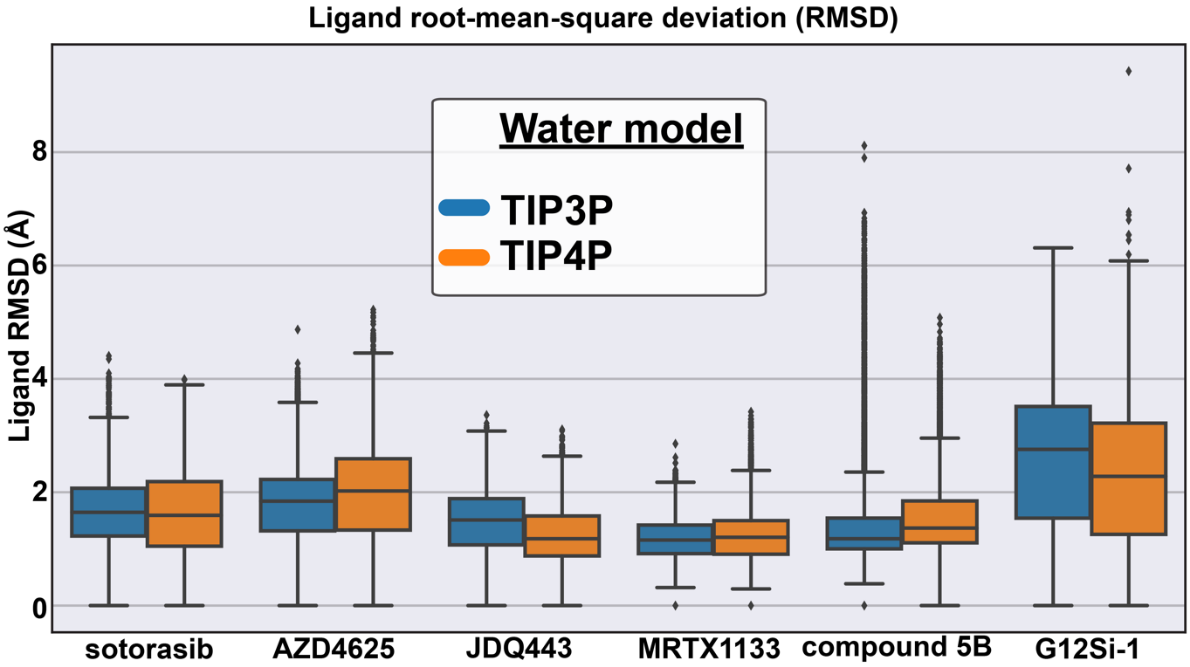

Figure S15. Ligand root-mean-square deviation (RMSD) in microsecond timescale MD simulations. Ligand heavy atom RMSD (ligand start conformation as the reference frame) was analyzed each 1 ns; boxplots consist of 10,000 data points (10 µs) for each system. Box displays the quartiles of the dataset (25–75%) and whiskers the rest of the data with maximum 1.5 IQR. Outliers are indicated with black diamonds.

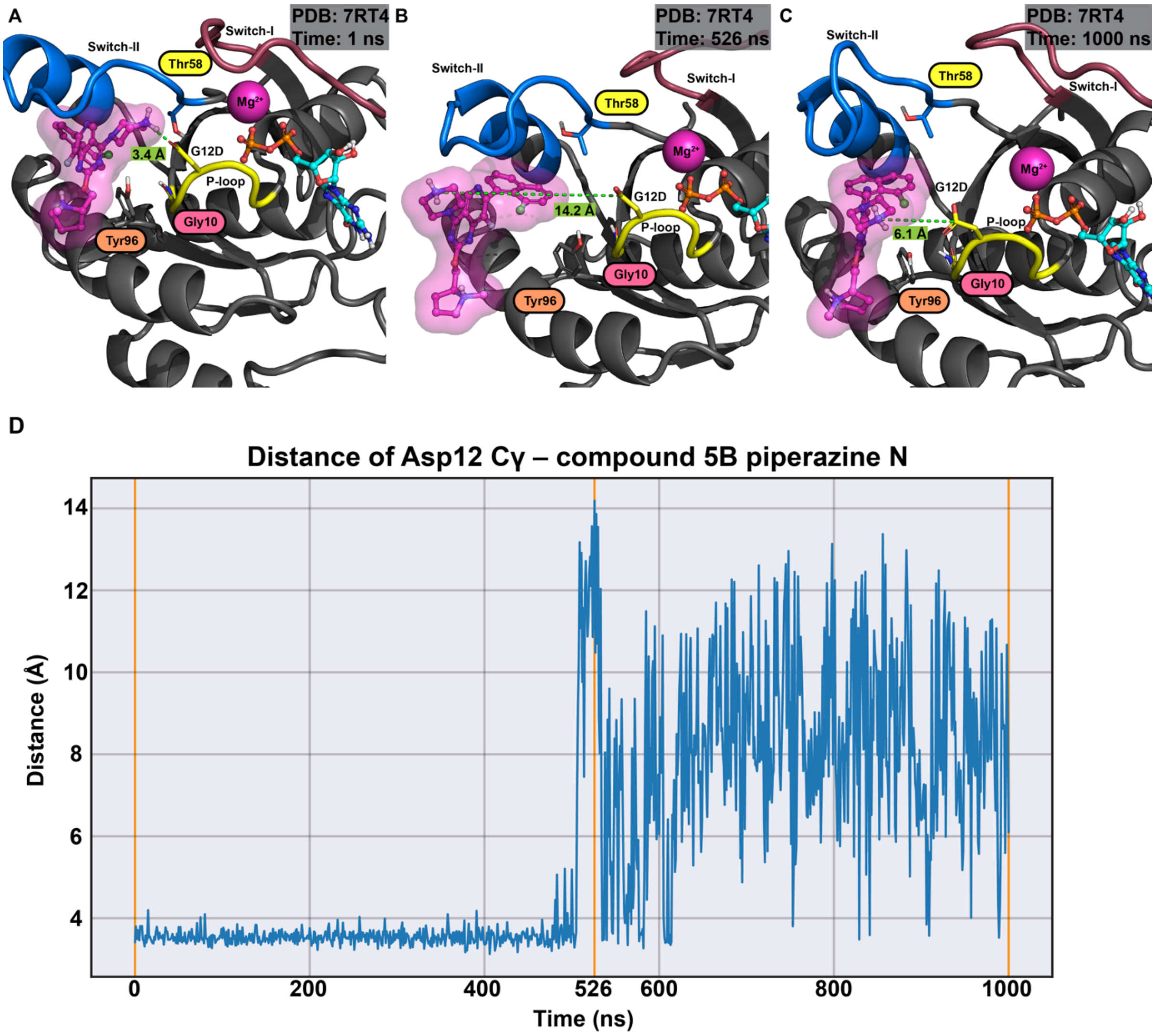

Figure S16. Compound 5B partially dissociates in one simulation replica next to the conserved water. Representative snapshots of the system (Compound5B_TIP3P_replica9) shown at (**A**) 1 ns, (**B**) 526 ns and (**C**) 1000 ns. (**D**) The dissociation of "compound **5B**" occurs after 500 ns of the simulation. The orange lines highlight the timeframes of the snapshots in **A–C**.

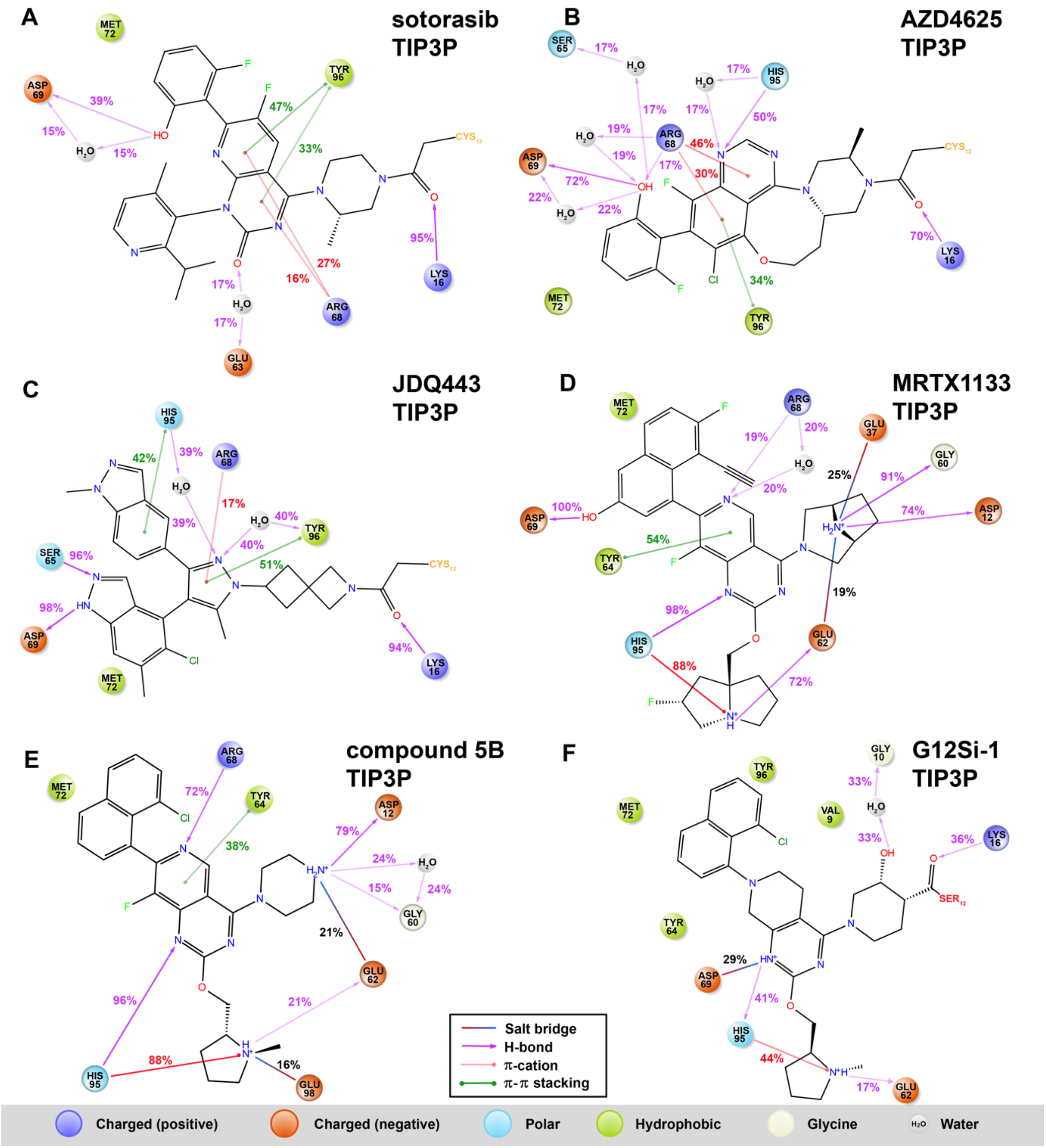

Figure S17. Protein–ligand interactions in TIP3P simulations. Interactions with >15% frequency are shown. Data consist of 10 µs for each individual system. The direct H-bond interaction to Gly10 of G12Si-1 (displayed in Figure 8) appears with 12% frequency, thus not shown here.

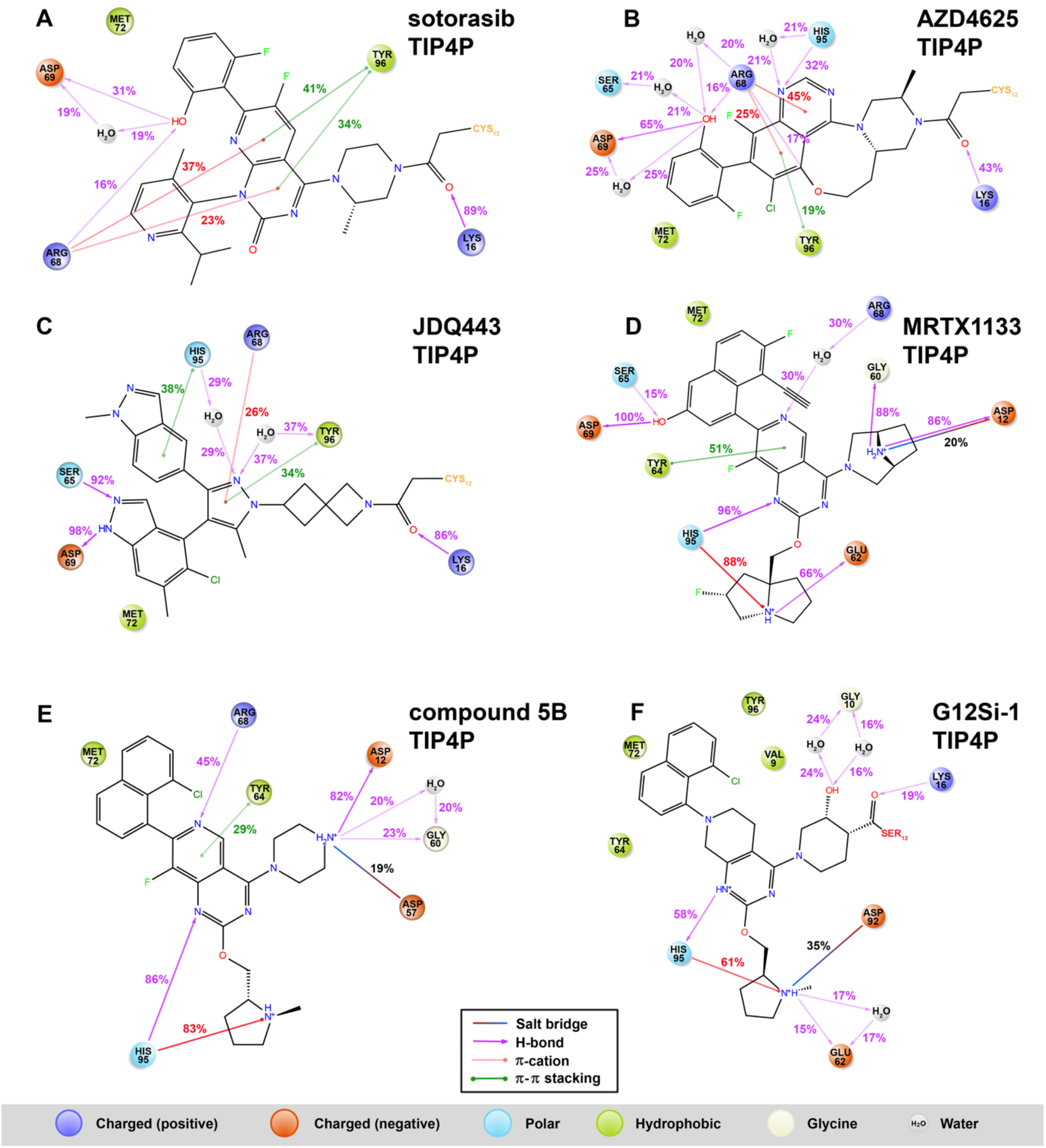

Figure S18. Protein–ligand interactions in TIP4P simulations. Interactions with >15% frequency are shown. Data consist of 10 µs for each individual system. The direct H-bond interaction to Gly10 of G12Si-1 (displayed in Figure 8) appears with 6% frequency, thus not shown here.

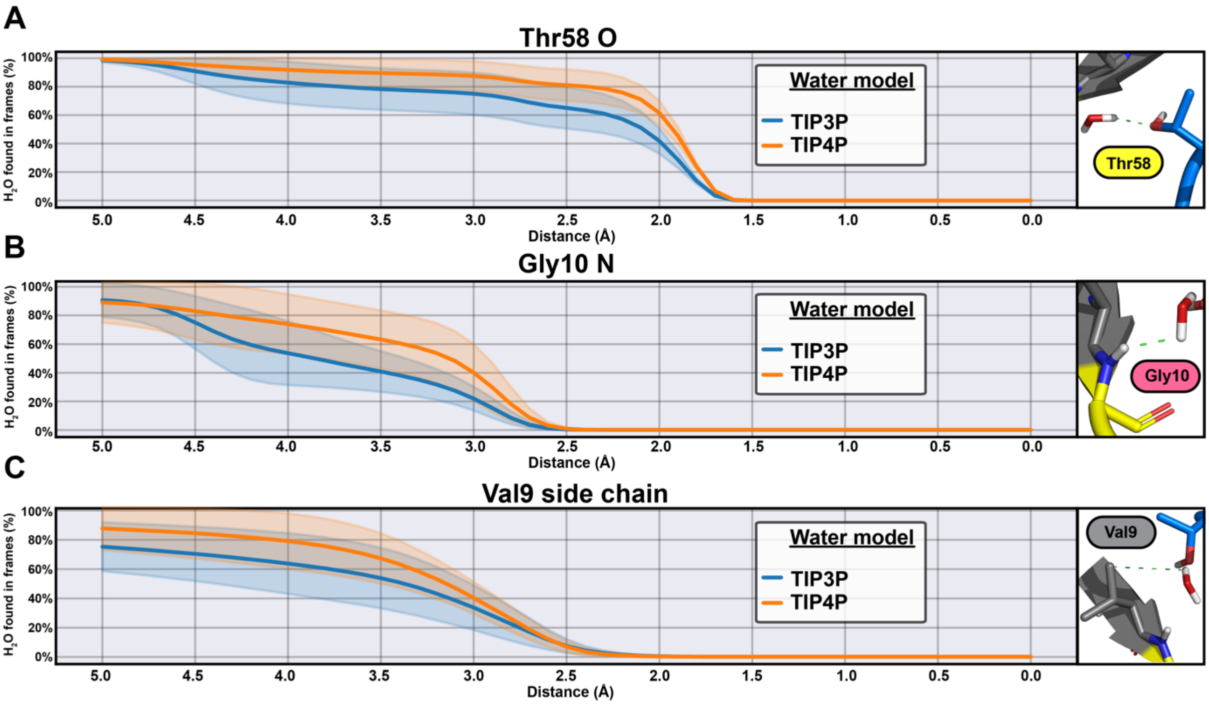

Figure S19. Discrepancy of observed water distances in TIP3P and TIP4P simulations. The line displays the average, and the shaded area represents SD. Data consist of 60 µs for each water model (all systems combined for the water models).
