# Supplementary material for: In Silico Evaluation of the Thr58-Associated Conserved Water with KRAS Switch-II Pocket Binders
Source: J Chem Inf Model. 2023 Feb 28;63(5):1490–505. doi: 10.1021/acs.jcim.2c01479 (PMC10015465; doi:10.1021/acs.jcim.2c01479)
Supplement: Supplementary file 3 — ci2c01479_si_003.pdf [file ci2c01479_si_003.pdf]

Supporting Information: Table S2

In silico Evaluation of the Thr58-associated Conserved Water with KRAS Switch-II Pocket Binders

*Renne Leini[1], Tatu Pantsar[1]**

[1]School of Pharmacy, Faculty of Health Sciences, University of Eastern Finland, Yliopistoranta 1C, 70210 Kuopio, Finland

*Email: tatu.pantsar@uef.fi

KEYWORDS. KRAS; water; WaterMap; molecular dynamics simulations; drug design.

Table S2. 2D-structures of the KRAS SII-P binders with publicly available structures.

KRAS	PDB ID	Ligand PDB ID	2D-structure
G12C	4LUC	20G	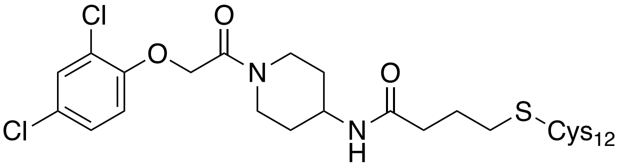
G12C	4LV6	20H	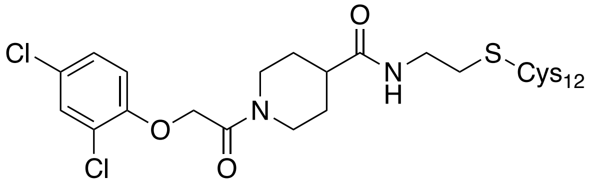
G12C	4LYF	21C	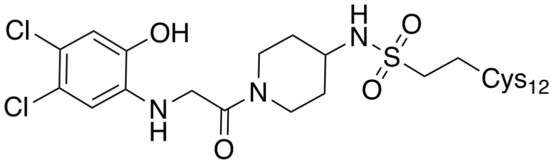
G12C	4M21	21Y	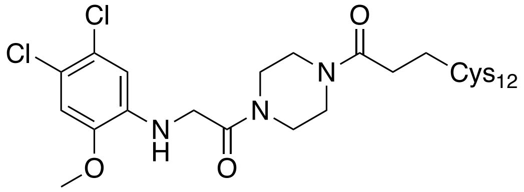
G12C	4LYJ	21F	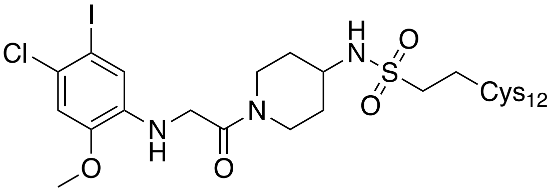
G12C	4M1O	21J	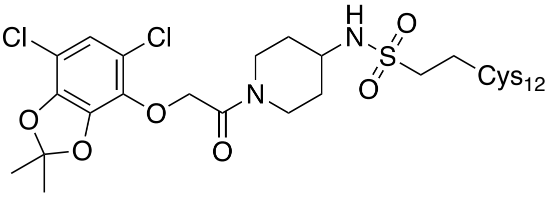
G12C	4M1S	21K	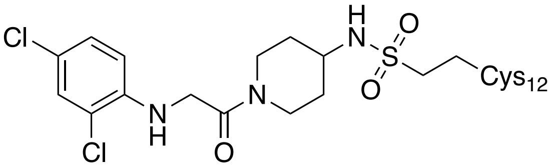
G12C	4M1Y	21S	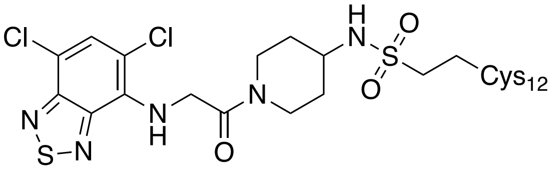
G12C	4M1W	21R	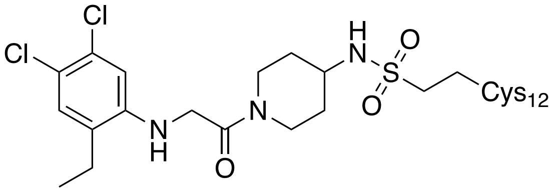

G12C	4M1T	21M	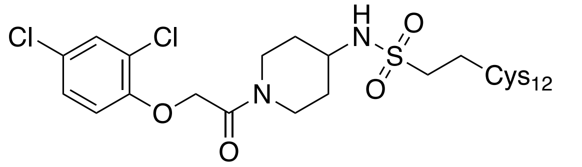
G12C	4M22	22C	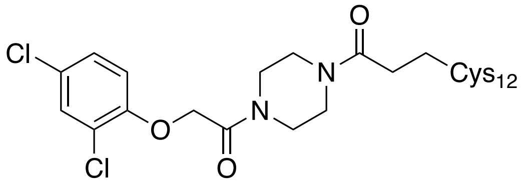
G12C	4LYH	21F	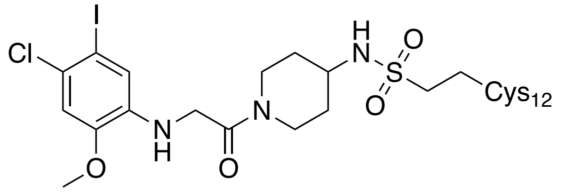
G12C	5F2E	5UT	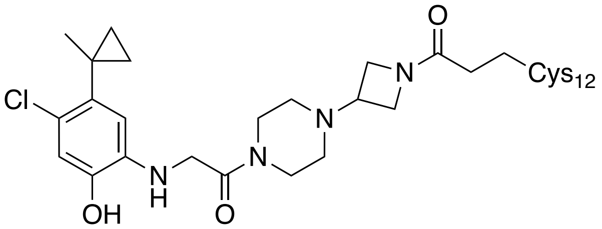
G12C	5V6S	8YD	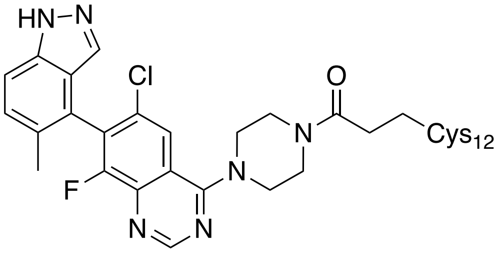
G12C	5V6V	8YA	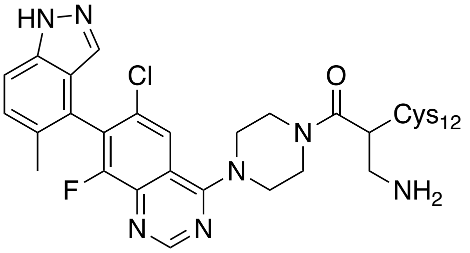
G12C	5V71	8ZG	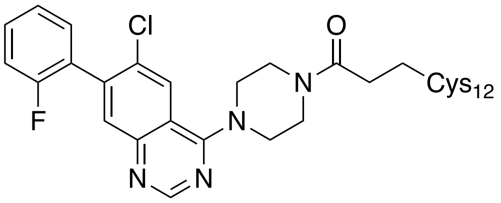

G12C	5V9L	91D	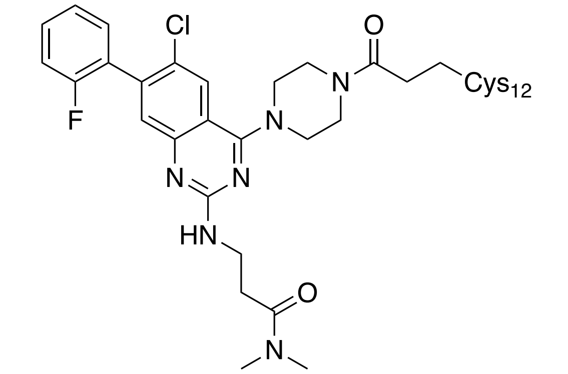
G12C	5V9O	91G	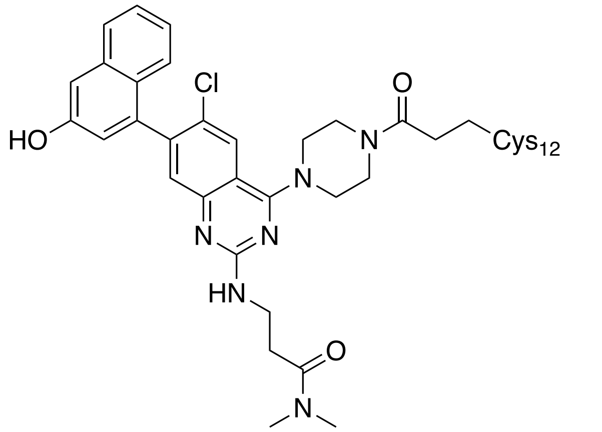
G12C	5V9U	91S	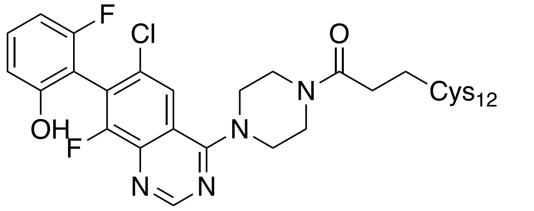
G12C	5YXZ	94C	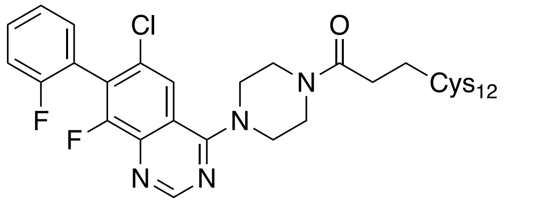
G12C	5YY1	94F	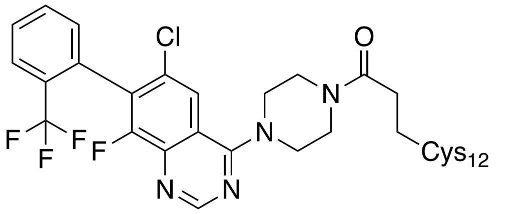
G12C	6B0V	C8G	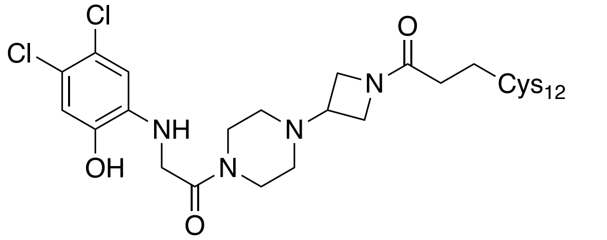

G12C	6B0Y	8ZG	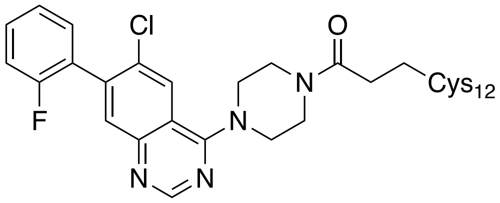
G12C	6N2J	K9M	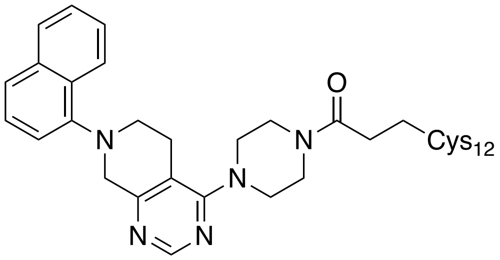
G12C	6N2K	K9J	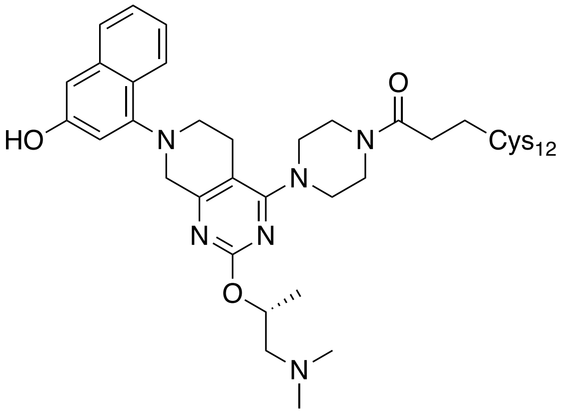
G12C	6OIM	MOV	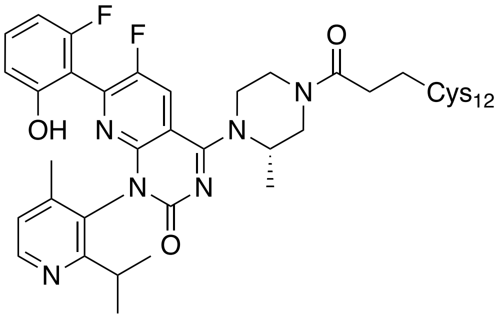
G12C	6P8W	O67	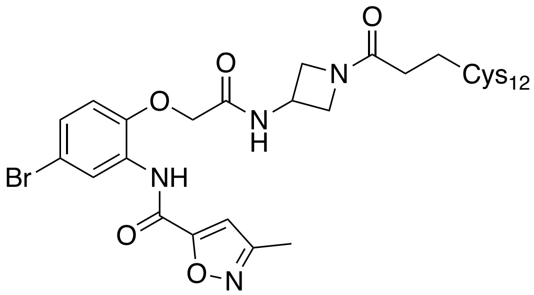

G12C	6P8X	O5V	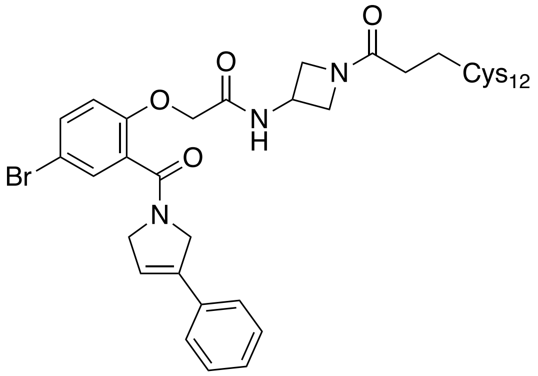
G12C	6P8Y	O5Y	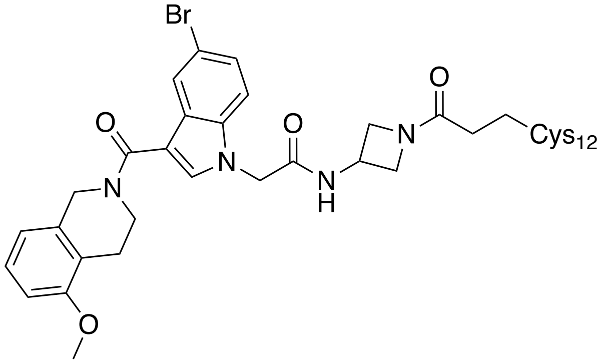
G12C	6P8Z	O5S	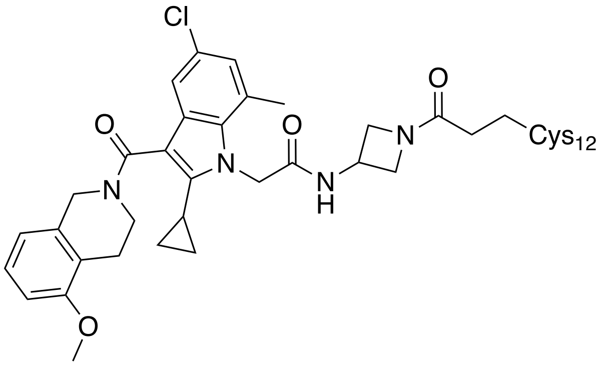
G12C	6PGO	OJ1	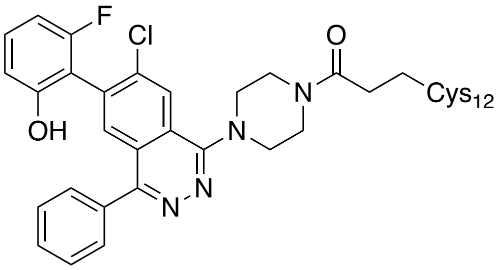
G12C	6PGP	OHY	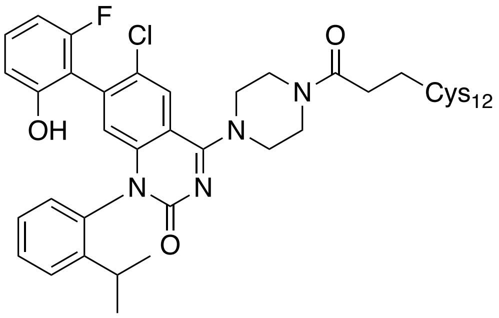

G12C	6T5B	O7K	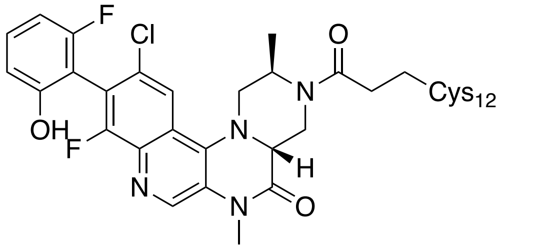
G12C	6T5U	MKW	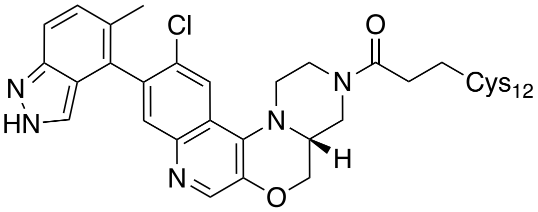
G12C	6T5V	MKZ	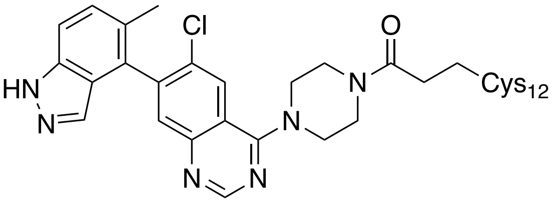
G12C	6TAM	MZQ	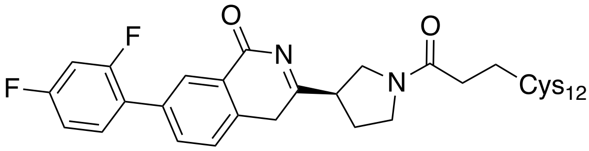
G12C	6TAN	MZN	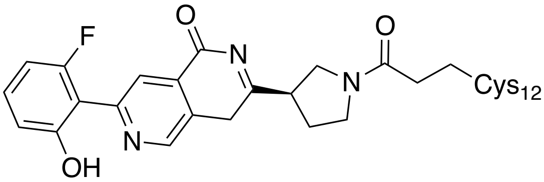
G12C	6USX	M1R	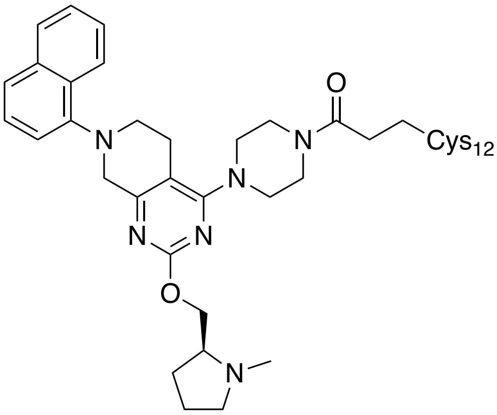

G12C	6UT0	M1X	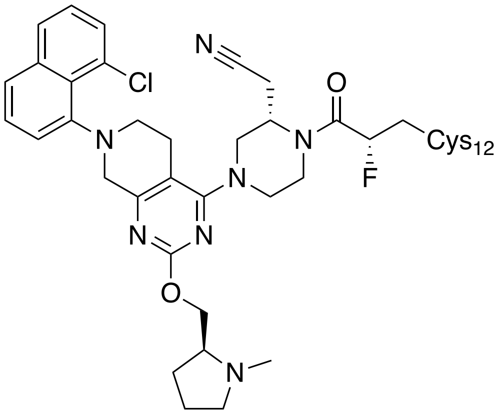
G12C	6USZ	QH4	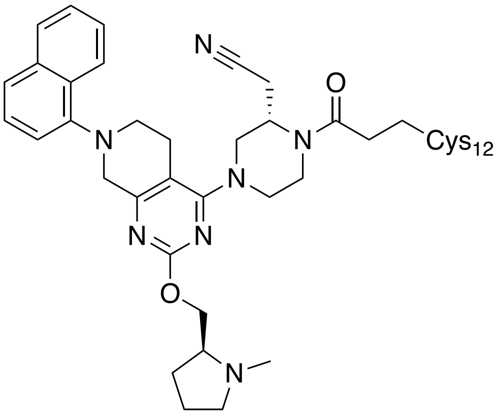
G12C	7MDP	Z07	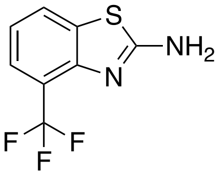
G12C	7RP3	MKZ	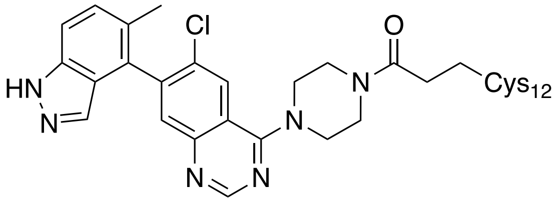
G12C	7RP4	MKZ	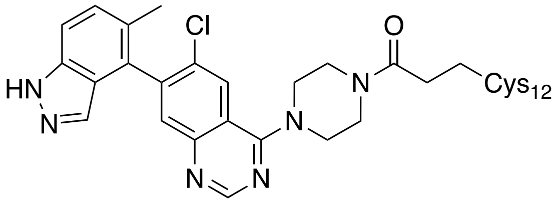
G12C	7R0M	H2T	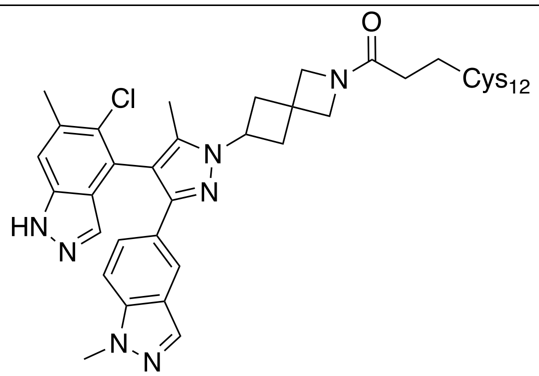

G12C	7R0N	H40	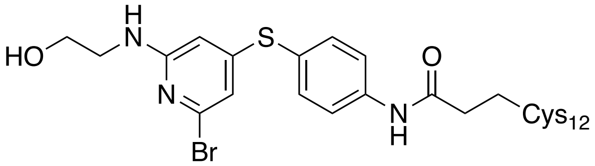
G12C	7R0Q	H0O	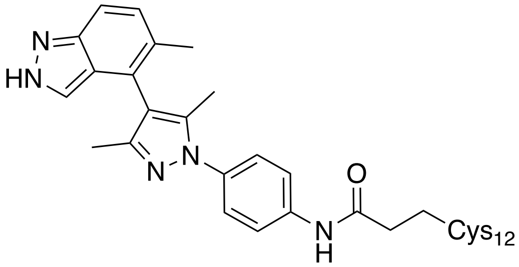
G12C	7O07	VLE	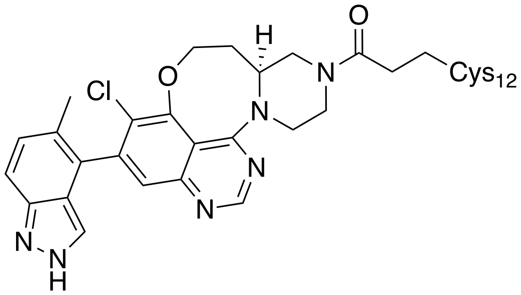
G12C	7O70	V4T	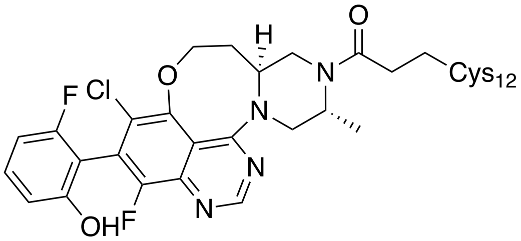
G12C	7O83	V52	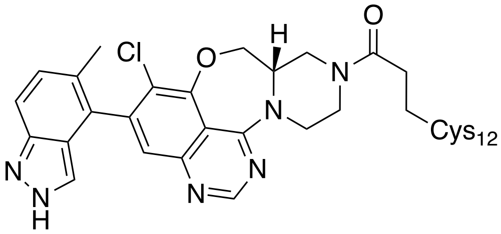
G12C	7YCC	IQC	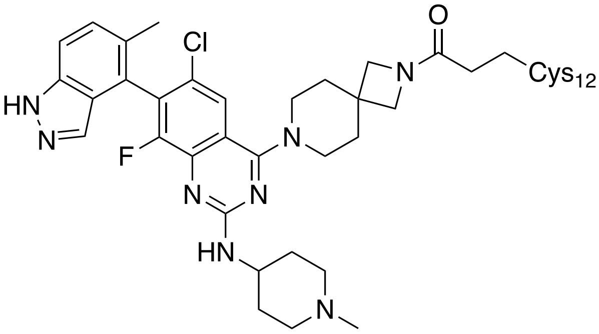

G12C	7YCE	IQN	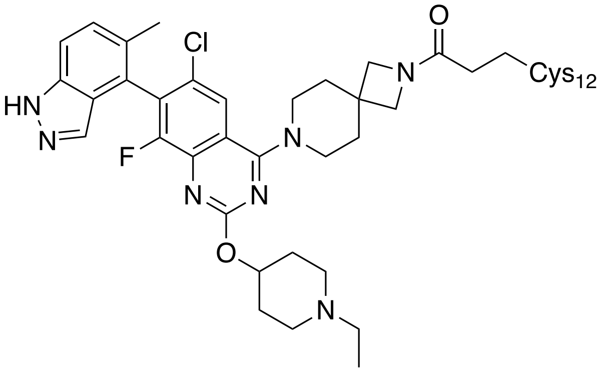
G12C	8DNI	U4L	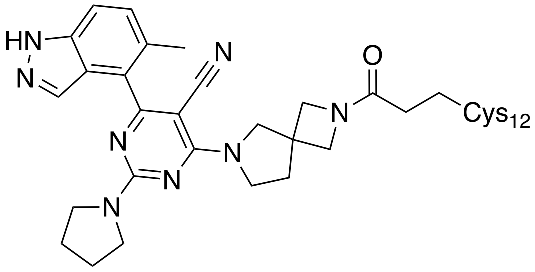
G12C	8DNJ	U4U	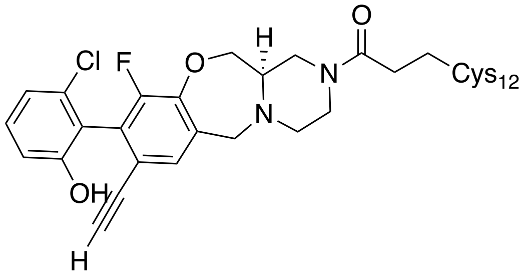
G12C	8DNK	U50	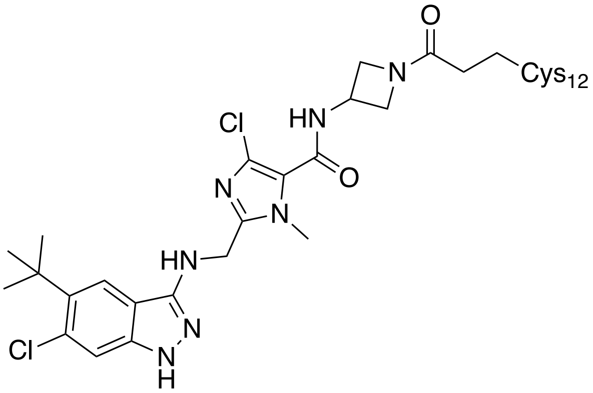
G12C	8AFC	LXK	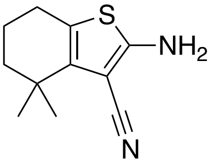

G12C	8AFB	LXD	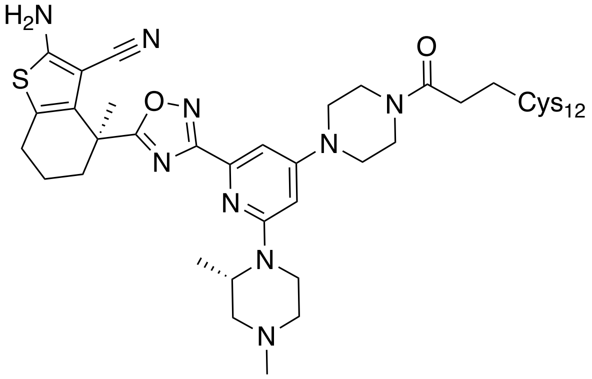
G12D	7RPZ	6IC	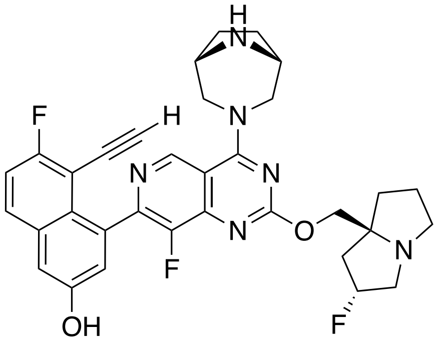
G12D	7RT1	7L8	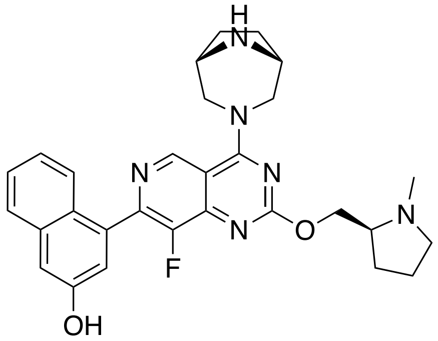
G12D	7RT2	7NL	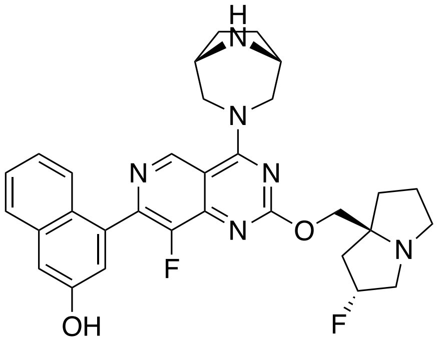
G12D	7RT3	7NZ	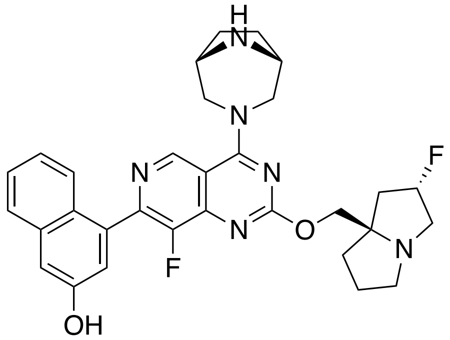

**G12D**	7RT4	7IZ	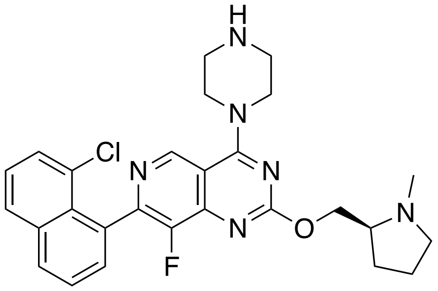
**G12D**	7RT5	7OE	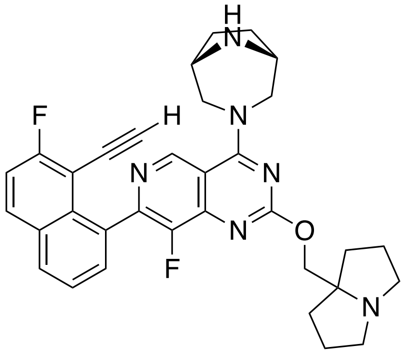
**G12D**	7T47	6IC	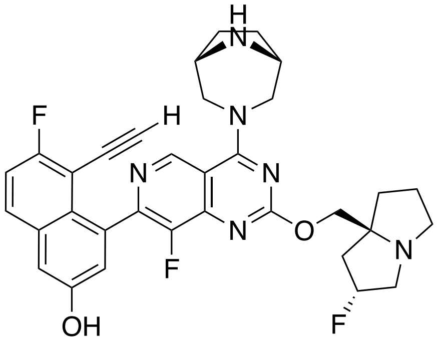
**G12D**	7EW9	05C	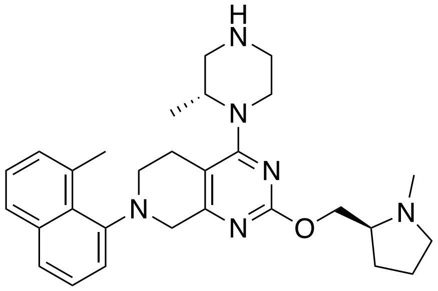
**G12D**	7EWA	05F	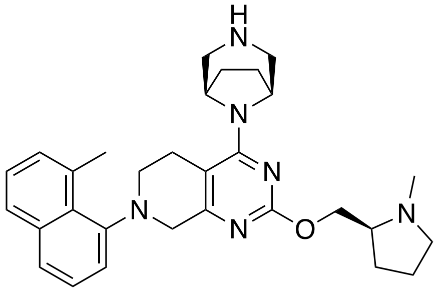

G12D	7EWB	05I	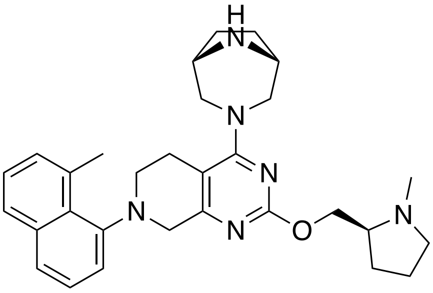
G12S	7TLE	I6T	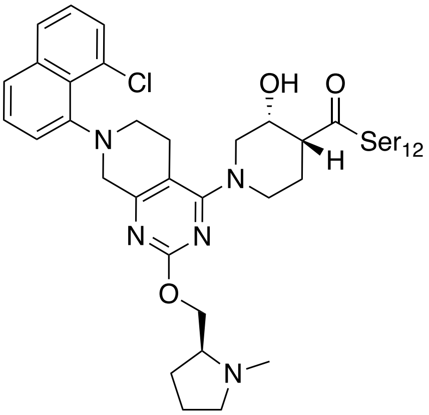
G12S	7TLG	I7H	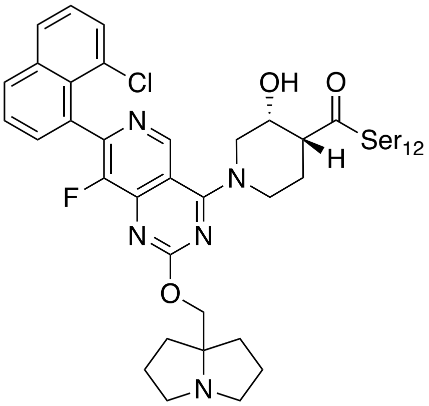
G12R	8CX5	P7U	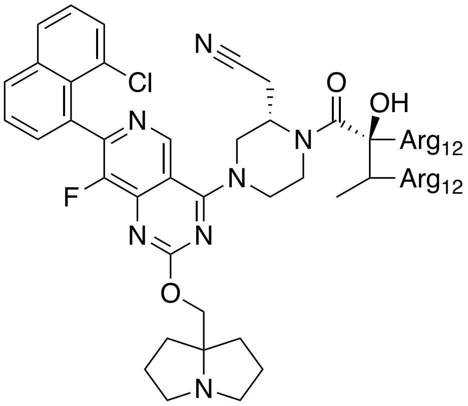
